# Transient and localized optogenetic activation of somatostatin-interneurons in mouse visual cortex abolishes long-term cortical plasticity due to vision loss

**DOI:** 10.1007/s00429-018-1611-7

**Published:** 2018-01-25

**Authors:** Isabelle Scheyltjens, Samme Vreysen, Chris Van den Haute, Victor Sabanov, Detlef Balschun, Veerle Baekelandt, Lutgarde Arckens

**Affiliations:** 1 Laboratory of Neuroplasticity and Neuroproteomics, KU Leuven, Naamsestraat 59, Box 2467, 3000 Leuven, Belgium; 2 Laboratory for Neurobiology and Gene Therapy, KU Leuven, 3000 Leuven, Belgium; 3 Laboratory of Biological Psychology, KU Leuven, 3000 Leuven, Belgium; 4 Leuven Viral Vector Core, KU Leuven, 3000 Leuven, Belgium

**Keywords:** Adulthood, Cortical plasticity, Multimodal, Somatostatin interneurons, Optogenetic stimulation, Dark exposure

## Abstract

Unilateral vision loss through monocular enucleation (ME) results in partial reallocation of visual cortical territory to another sense in adult mice. The functional recovery of the visual cortex occurs through a combination of spared-eye potentiation and cross-modal reactivation driven by whisker-related, somatosensory inputs. Brain region-specific intracortical inhibition was recently recognized as a crucial regulator of the cross-modal component, yet the contribution of specific inhibitory neuron subpopulations remains poorly understood. Somatostatin (SST)-interneurons are ideally located within the cortical circuit to modulate sensory integration. Here we demonstrate that optogenetic stimulation of visual cortex SST-interneurons prior to eye removal decreases ME-induced cross-modal recovery at the stimulation site. Our results suggest that SST-interneurons act as local hubs, which are able to control the influx and extent of cortical cross-modal inputs into the deprived cortex. These insights critically expand our understanding of SST-interneuron-specific regulation of cortical plasticity induced by sensory loss.

## Introduction

The adult mammalian brain retains the intrinsic capacity to recover from sensory deprivation, even long after critical periods of enhanced plasticity for sensory cortical development are closed. In human patients, late-onset vision loss can for example be compensated by enhanced tactile acuity (Norman and Bartholomew 2011), or improved sound localization abilities (Voss et al. 2004; Fieger et al. 2006), which involve the recruitment of the visual cortex during non-visual performance (Siuda-Krzywicka et al. 2016). We recently showed in a mouse model of one-eyed vision, due to monocular enucleation (ME) induced at P120, how the originally deprived visual cortex displays a strong reactivation over a period of 7 weeks. This functional recovery relied not only on the potentiation of spared-eye inputs in the binocular zone in the first weeks post-ME, but clearly also on ensuing somatosensory whisker-related activation of especially the medial monocular zone of the visual cortex (Van Brussel et al. 2011; Nys et al. 2014).

Inhibitory neurotransmission is a central player in regulating different aspects of experience-dependent and deprivation-induced cortical plasticity (Feldman 2000; Baroncelli et al. 2011; Keck et al. 2011; Nys et al. 2015). The strength, maturation and functional recruitment of inhibitory neurotransmission gradually increase from development into adulthood, and the concurrent age-dependent modulation of the excitatory drive permits—or puts a brake on—experience-driven plasticity during specific age-windows (Fagiolini and Hensch 2000; Bavelier et al. 2010; Nys et al. 2014). In the well-studied field of ocular dominance plasticity following reversible monocular deprivation by lid-suture, a dark exposure (DE)-pretreatment-strategy rejuvenates the inhibitory neurotransmission by reinstating immature inhibitory characteristics in the binocular visual cortex, including a high GABA release probability, a decreased paired-pulse depression and the re-expression of endocannabinoid-dependent inhibitory long-term depression (Huang et al. 2010). DE is thus a proven strategy to reopen critical period-like windows of unimodal ocular dominance plasticity in the binocular cortex of adult rodents (He et al. 2007). Its use in the context of ME, a more severe and permanent visual deficit strategy compared to lid-suture, also alters the level of cortical inhibition, albeit in opposite directions for the binocular compared to the medial monocular visual cortex. In the ME model, DE results in a local increased inhibitory tone and the absence of cross-modal, whisker-driven, recovery of the medial monocular cortex (Nys et al. 2015; Huang et al. 2015).

Based on these studies it remains unclear if such brain region-specific changes in inhibitory tone correlate with specific inhibitory cell types, and how they each affect the outcome of the recovery profile of the sensory deprived visual cortex. Although non-invasive interventions such as DE could be easily translatable to human patients and may have a strong therapeutic potential in reinstating cortical plasticity when and where it is desirable (Hofer et al. 2006) or block it when maladaptive (Meredith et al. 2012; Sharma and Glick 2016), knowledge on which particular inhibitory interneuron type is locally involved may help refine such interventions targeted towards recovery from loss of sensory function.

Somatostatin (SST-) interneurons are centrally located within the cortical network and contribute to critical period-like ocular dominance plasticity in the binocular cortex following monocular lid-suture (Fu et al. 2014, 2015; Tang et al. 2014). SST-interneuron-specific features made us hypothesize they also play a role in the reactivation strategies of the adult visual cortex following ME, with an emphasis on the cross-modal recruitment based on whisker-related inputs, suggested to mainly be supported by corticocortical connections (Van Brussel et al. 2011; Iurilli et al. 2012). Adult SST-interneurons are mainly activated by intracortical inputs (Beierlein 2003; Cruikshank et al. 2007, 2010) and lack direct thalamic input in contrast to parvalbumin (PV)-interneurons. SST-interneurons target distal dendrites of pyramidal neurons (Kawaguchi and Kondo 2002; Silberberg and Markram 2007; Kapfer et al. 2007; Murayama et al. 2009) where they could modulate (multi-sensory) integration (Lee et al. 2013; Yang et al. 2016). A large population of SST-interneurons, the Martinotti cells, reside abundantly in infragranular layers where the impact of cross-modal invasion in the deprived visual cortex following ME was shown to be most pronounced (Van Brussel et al. 2011).

To examine if SST-interneuron-activity can up- or downregulate the level of reactivation of the visual cortex upon contralateral vision loss by ME, we applied optogenetic stimulation of SST-interneurons in both monocular and binocular V1. DE prevents cross-modal plasticity when applied just prior to the ME procedure (Nys et al. 2015). The ongoing state of inhibitory neurotransmission around the onset of sensory loss thus seems to determine how the cortex ultimately responds in terms of functional recovery. We therefore optogenetically stimulated SST-interneurons in this same time frame, immediately prior to ME. We judged the long-term reactivation profile of binocular and monocular visual cortex using a well-established approach, based on cortical *zif268*-mRNA expression levels, and compared it to ME-only mice or ME mice that also received a DE-treatment prior to ME. The finding of a lowered long-term reactivation of the visual cortex following such a short-term SST-interneuron prestimulation indicates a central regulating role for SST-interneurons in experience-dependent cortical plasticity particularly at deprivation onset. Comparison to ME mice in which the same stimulation paradigm was applied half-way the recovery period, at the time point when the cross-modal phase of recovery previously became apparent (Van Brussel et al. 2011), revealed a similar but milder phenotype. We also combined the two pretreatment-strategies by stimulating SST-interneurons during the DE period to assess whether SST-interneuron activity impinges on the same underlying mechanisms to modulate cross-modal plasticity compared to the inhibitory alterations caused by DE, or rather that both strategies work independently in the same direction, and that when combined in time, serve as an almost complete off-switch for cross-modal cortical plasticity.

## Materials and methods

### Animals

SST-Cre knock-in mice (*n* total = 39) and SST-Cre mice crossed with an eYFP-reporter strain (*n* = 6) of either sex were obtained from The Jackson Laboratory (Sacramento, CA, USA) (SST-Cre mice: STOCK Sst^tm2.1(cre)Zjh^/J, Stock No 013044; eYFP-reporter mice: Gt(ROSA)26Sor^tm1(EYFP)Cos^, Stock No 006148) and were housed under standard laboratory conditions under an 10/14-h dark/light cycle with food and water available *ad libitum*. All experiments were approved by the ethical committee of the KU Leuven and were in strict accordance with the European Communities Council Directive of 22 September 2010 (2010/63/EU) and with the Belgian legislation (KB of 29 May 2013). Every effort was made to minimize animal suffering and to reduce the number of animals.

### Viral vectors

The viral vectors (rAAV2/7-CMV-Flex-SSFO-mCherry and rAAV2/7-CMV-flex-eGFP) were produced at the Leuven Viral Vector Core as previously described (Van der Perren et al. 2011). We used the recombinant adeno-associated viral vector (rAAV) 2/7 containing the inverted terminal repeats (ITRs) of rAAV2, and the capsid of rAAV7, because this serotype results in a proper expression pattern within the region of interest, i.e. the primary visual cortex (V1), without spreading into other nearby sensory areas (Scheyltjens et al. 2015). The rAAV2/7 viral vectors contain the cytomegalovirus promoter (CMV) to achieve strong expression in the mouse cortex and carry the transgene, the stable-step function opsin [SSFO, C128S/D156A mutant of Channelrhodopsin-2 (ChR2)] between two pairs of incompatible lox-sites, resulting in a Cre-dependent transcription mechanism (Vandeputte et al. 2014). For detection purposes, the SSFO-gene is fused to a gene encoding the fluorescent protein mCherry. The genomic titer corresponds to 8.55 × 10^11^ genome copies (GC) per milliliter. The sham-control vector is similar to the SSFO-vector, but carries an enhanced green fluorescent protein (eGFP) gene instead of the SSFO-mCherry gene. The genomic titer of this viral vector corresponds to 1.55 × 10^12^ GC/mL.

Viral vector injections were performed in SST-Cre mice crossed with an eYFP-reporter strain to verify cell type-specific SSFO-expression (*n* = 3, P90) and to perform patch clamp whole-cell recordings to assess the functionality of the SSFOs (*n* = 3, P70). For the in vivo optogenetic stimulation experiments, viral vector injections were performed in adult SST-Cre mice (P90). All animals were anesthetized by intraperitoneal injections of a mixture of ketamine hydrochloride (75 mg/kg; Dechra Veterinary Products; Eurovet, Bladel, The Netherlands) and medetomidine hydrochloride (1 mg/kg; Orion; Janssen Animal Health, Beerse, Belgium). Vectors were stereotactically injected through a small craniotomy into the primary visual cortex (V1) of the left hemisphere: 3.2 mm posterior to bregma, 2.5 mm lateral to the midline, at a depth of 400 µm from the surface (Paxinos and Franklin 2012). Per injection a volume of 200 nL (in steps of 13.8 nL every 30 s) of viral vector was delivered via a glass capillary (~ 20 µm tip diameter) using a Nanoject II Auto-Nanoliter Injector (Drummond Scientific, Broomall, PA). Within the same craniotomy, three injections of 200 nL approximately 300 µm apart were performed in a triangular configuration to result in a total injected volume of 600 nL per mouse. After each injection, the capillary was left in place for an additional 2 min before being slowly retracted. Following wound suturing, the anesthesia was reversed by intraperitoneal injection of atipamezol hydrochloride (1 mg/kg; Orion; Elanco Animal Health, Antwerp, Belgium).

### Verifying the specificity and expression pattern of the viral vector transduction

A subset of SST-Cre mice crossed with an eYFP-reporter strain (*n* = 3) were injected to express SSFO in SST-interneurons and were verified for specificity. Four weeks post-injection the animals were deeply anesthetized by intraperitoneal injection of sodium pentobarbital (Nembutal, 600 mg/kg, Ceva Sante Animale, Brussels, Belgium) and were transcardially perfused with 1% paraformaldehyde in phosphate-buffered saline (PBS). Subsequently, the brains were removed and postfixed over a period of 24 h in paraformaldehyde (4%) in PBS. Fifty micrometers free-floating serial sections were prepared on a Vibratome (Microm HM 650 V, Thermo Scientific, Walldorf, Germany). Specific expression in SST-interneurons was verified by means of mCherry—eYFP double stainings. Polyclonal rabbit anti-red fluorescent protein (RFP) (Rockland Immunochemicals, Limerick, PA, ab600-401-379; 1:5000) was used to amplify mCherry fluorescence, and polyclonal chicken-anti-eGFP (Abcam, Cambridge, UK, ab13970; 1:2000) was used to amplify the eYFP signal. As secondary antibodies Alexa Fluor 594 (polyclonal goat anti-rabbit IgG, Life Technologies, Ghent, Belgium, A11011 1:250) and DyLight 488 (polyclonal goat anti-chicken IgY, Abcam, ab96947; 1:250) were used. After washing the sections in PBS and incubation with normal goat serum (Merck Millipore, Overijse, Belgium) for 45 min, the sections were incubated over a period of 24 h with primary antibody diluted in Tris-NaCl blocking buffer (TNB). After rinsing in PBS the sections were incubated with the secondary antibodies diluted in TNB for 2 h and were then counterstained with DAPI (2 µL/100 mL PBS, Sigma-Aldrich, St. Louis, MO, 3260) before mounting and cover slipping with Mowiol solution. Overview images of the injection sites were made with an inverted FV1000 confocal microscope (IX81, Olympus, Aartselaar, Belgium) using a 20× objective (NA 0.75) at a resolution of 512 × 512. Pictures were taken as *z*-stacks at 3-µm intervals covering the entire thickness of the section (50 µm). Higher magnification images were made with a 60× objective (NA: 1.35) at a resolution of 512 × 512, again as *z*-stacks at 3-µm intervals. Image acquisition was done with FluoView10 software (Olympus).

### Patch clamp whole-cell recordings to validate the functionality of the SSFOs

After verifying cell type-specific expression of SSFOs to SST-interneurons, a second set of SST-Cre mice crossed with the eYFP-reporter strain (*n* = 3, P70) were prepared for patch clamp whole-cell recordings in current clamp mode, 4 weeks post-injection with the viral vector and expressing SSFOs in SST-interneurons. The mice were sacrificed by cervical dislocation, decapitated and brains were quickly transferred to ice-cold oxygenated (95% O_2_, 5%CO_2_) artificial cerebrospinal fluid (aCSF in mM: 124 NaCl, 4.9 KCl, 25.6 NaHCO_3_, 1.2 NaH_2_PO_4_; 2 MgSO_4_, 10 glucose, pH 7.3–7.4). The visual cortex was coronally sliced (400 µm thick) on a Vibratome (Microm HM 650 V, Thermo Scientific) in ice-cold oxygenated aCSF. Cortical slices were allowed to recover in an interface chamber filled with oxygenated aCSF solution at RT for at least 1 h. Then, individual slices were placed in a recording chamber and superfused continuously at a rate of 2.5 mL/min with aCSF at 32 °C. To prevent activation of SSFOs by ambient light, all steps were performed in a light-protected environment. Whole-cell recordings from mCherry-expressing SST-interneurons were performed in current clamp mode using a patch-clamp amplifier (MultiClamp 700B, Axon Instruments, New York, NY). All data were low-pass filtered at 2 kHz and acquired at 10 kHz with the Digidata 1440 data acquisition system and pClamp 10.1 software (Molecular Devices, Sunnyvale, CA). Patch microelectrodes were filled with a solution containing (in mM): 135 K-gluconate, 5 MgCl2, 10 K-Hepes, 20 glucose; pH 7.25. Pipette resistance was 3–4 MΩ. Patching was performed under visual control by an infrared differential interference contrast optics system (Axioskop2 FC Plus, Zeiss Instruments, Jena, Germany). For the visualization of mCherry fluorescence and the optical stimulation the LED KSL 70 (Rapp OptoElectronic, Hamburg, Germany) and Lambda DG-4 Plus Illumination system (Sutter Instrument, Novato, CA) were used. To activate SSFOs, 470 nm light pulses (1 s, ~ 0.5–1 mW/mm^2^) were given, followed 5 s later by a 590 nm light pulse (2 s, ~ 0.5–1 mW/mm^2^) to deactivate them.

### Chronic implantation for light-delivery

**Fig. 1:**
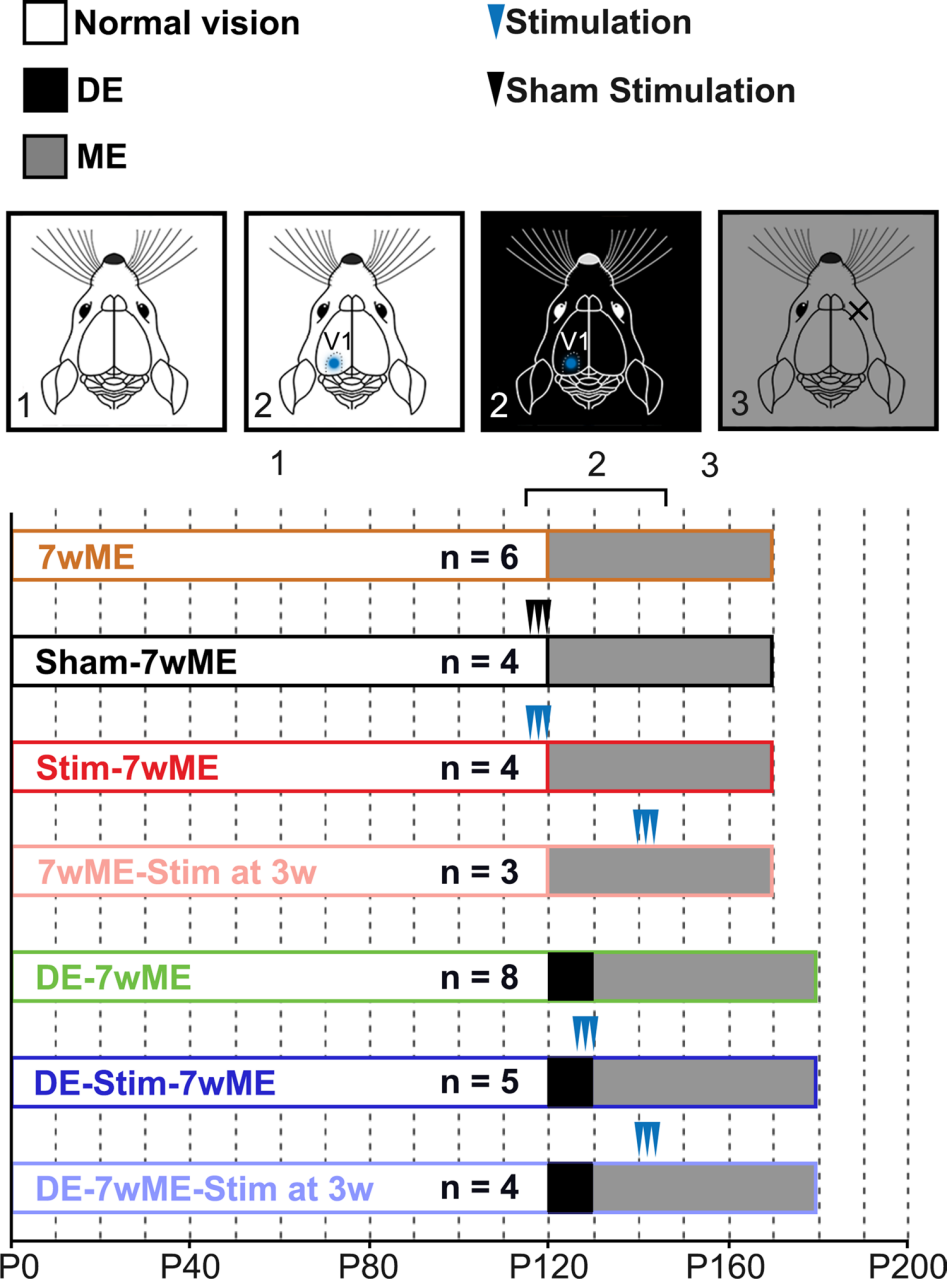
Experimental setup of the different optogenetically stimulated and control conditions. All animals underwent a period of normal vision until around P120 (1). Optogenetic stimulation of normally sighted and dark exposed animals was performed in V1 (blue dot, 2) on 3 consecutive days (three arrow heads), twice a day in the left hemisphere, prior to contralateral ME (3), or at 3 consecutive days at 3 weeks post-ME

After verification of successful activation and deactivation of SSFOs in SST-interneurons, a different set of SST-Cre mice was used for the in vivo SST-interneuron stimulation experiments. Three weeks following viral vector injection, Sham-7wME, Stim-7wME, 7wME-Stim at 3w, DE-Stim-7wME and DE-7wME-Stim at 3w mice (Fig. 1) were equipped with a headpiece implant (Doric Lenses, Quebec, Canada) for light delivery to chronically stimulate the SSFOs. The SSFO-vector injected mice were anesthetized with a carbogen–isoflurane mixture with a flow rate of 1–1.5 L/min. Anesthesia was induced with 4% isoflurane (Iso-Vet 1000 mg/g, Eurovet) and maintained with 1–1.5% isoflurane. The mice were positioned in a stereotaxic apparatus on a heating blanket and the eyes were protected from dehydration with ointment (Fucithalmic 10 mg/g, Amdipharm, London, UK). Following resection of the disinfected skin and hydrating the skull with saline (0.9% NaCl), the pre-existing craniotomy made for viral vector injections was protected with silicon sealant (Kwik-Cast, World Precision Instruments, Sarasota, FL) to protect the brain tissue from cleaning the skull with 3% H_2_O_2_ and corroding it slightly with Etch Gel (Henry Schein Dental, Vilvoorde, Belgium) to allow a stable and long-term grip for the dental cement. To strengthen the grip of the dental cement further, a thin layer of primer (Kerr/Hawe Optibond FL, Bioggio, Switzerland) and UV-curable adhesive (Kerr/Hawe Optibond FL) was applied to the dry skull. The mono fiber-optic cannula with magnetic receptacles (Doric Lenses) was positioned such that the optic fiber is located precisely above the craniotomy. The blunt-end optic fiber protruding 0.5 mm from the magnetic receptacle is not inserted directly into the brain, which could cause tissue damage, but is positioned on top of the brain surface. The head-implant was next secured in this position with UV-curable dental cement (Tetric Evoflow, Ivoclar Vivadent, Hoofddorp, The Netherlands) and was left in place during all further experimental procedures. After fixing the skin with tissue adhesive (3M Vetbond, 3M, St. Paul, MN), the animals were administered meloxicam (1 mg/kg; Boerhinger Ingelheim, Alkmaar, The Netherlands) subcutaneously as a systemic analgesic and were allowed to recover from the anesthesia on a heating pad.

### SSFO-stimulation protocol in freely moving animals

One to two weeks following surgery, the animals (Sham-7wME, Stim-7wME, 7wME-Stim at 3w, DE-Stim-7wME and DE-7wME-Stim at 3w, Fig. 1) were transferred into a light-controlled stimulation box and received blue-light pulses two times a day in order to expose the animals to regular activation of SST-interneurons, immediately prior to (Sham-7wME, Stim-7wME, DE-Stim-7wME), or 3 weeks after (7wME-Stim at 3w, DE-7wME-Stim at 3w) the enucleation procedure. Sham-7wME, Stim-7wME, 7wME-Stim at 3w and DE-7wME-Stim at 3w animals were kept under a normal light/dark cycle and were stimulated in a light environment. To investigate whether SST-interneuron activation through SSFOs could change the response to DE and subsequent ME, the DE-Stim-7wME animals were optogenetically stimulated in complete darkness during the last 3 days of the DE period, during which the animals were kept in complete darkness during 10 consecutive days (Fig. 1). This strategy was chosen based on previous literature showing that pharmacological manipulations during the last 3 days of DE could successfully undo DE-induced changes relating to the inhibitory neurotransmission (Huang et al. 2010). For blue light delivery, the magnetic receptacle was coupled into an optic fiber connected through a rotary joint to a DPSS-laser source with a wavelength of 473 nm (Shanghai Laser & Optics Century Co., Ltd., Shanghai, China) and passing through a 50% attenuator (Doric Lenses) to achieve a stable laser output at the optic fiber tip with an intensity of 70 mW/mm^2^. The optic fiber has a core diameter of 200 µm, an outer diameter of 245 µm and a numerical aperture of 0.37. Taking these parameters into consideration, as well as the light scattering when passing through brain tissue, the approximate light intensity ranges from 56 mW/mm^2^ to 740 µW/mm^2^ at depths ranging from 0 to 1 mm from the cortical surface (calculated with the online predictor of irradiance values in the mammalian brain: http://web.stanford.edu/group/dlab/cgi-bin/graph.chart.php). These intensities still allow activation of the SSFO because they remain sensitive to laser intensities of a few mW/mm^2^ to as low as 10 µW/mm^2^ (Yizhar et al. 2011). The chosen stimulation paradigm was consistent among all experimental animals, was performed on 3 consecutive days prior to ME, or on 3 consecutive days at 3 weeks post-ME and consisted of two stimulation trains of 1 h, with an 8 h interval. Each train consisted of four 2-s laser pulses with an interval of 15 min between two consecutive laser pulses. This stimulation paradigm was chosen to achieve chronic hyperexcitability at regular time points of SST-interneurons without desensitizing them by continuous stimulation and is comparable to stimulation paradigms described previously in literature proven to successfully activate SSFOs (Sidor et al. 2015).

### Visual deprivation paradigm and tissue preparation

Animals were monocularly enucleated as described previously (Aerts et al. 2014) (Fig. 1). Briefly, the animals were anesthetized by intraperitoneal injection of a mixture of ketamine hydrochloride (75 mg/kg; Dechra Veterinary Products; Eurovet) and medetomidine hydrochloride (1 mg/kg; Orion; Janssen Animal Health). The right eye, contralateral to the injection site, was carefully removed, and the orbit was filled with hemostatic cotton wool (Qualiphar, Bornem, Belgium) in case of bleeding. Anesthesia was reversed with atipamezol hydrochloride (1 mg/kg; Orion; Elanco Animal Health) and the animals were allowed to recover on a heating pad. Following enucleation, the mice were housed in their home cages under standard 10/14-h dark/light conditions for a 7-week survival period.

To interpret the *zif268*-mRNA related activity pattern 7 weeks after ME, the mice were placed overnight in their home cages in a dark room to reduce *zif268*-expression to basal levels. The following day the mice were placed in a high-light environment to upregulate visually driven *zif268*-mRNA expression. A parallel transfer to new cages induces cage exploration for somatosensory driven *zif268*-mRNA expression. After 45 min, when experience-driven *zif268*-mRNA expression is maximally upregulated, the mice were killed by cervical dislocation. The brains were rapidly extracted and immediately frozen in 2-methylbutane (Merck, Overijse, Belgium) at a temperature of − 40 °C and stored at − 80 °C until sectioning. For in situ hybridization (ISH) experiments, 25 µm-thick sections were prepared on a cryostat (HM 500 OM, Microm, Thermo Scientific), mounted on 0.1% poly-l-lysine-coated (Sigma-Aldrich) slides, and stored at − 20 °C until further processing.

### In situ hybridization for *zif268*-mRNA

Adult mice have the potential to recover from severe loss of sensory input as is evidenced by the strong reactivation of the monocular visual cortex 7 weeks after unilateral enucleation. By means of radioactive in situ hybridization (ISH) for the activity marker *zif268* it was previously successfully shown that increased *zif268*-mRNA levels coincide with increased activation potential of the cortex which can generally be subdivided in two windows: early visually driven recovery where the binocular zone expands, and cross-modal plasticity between 3 and 7 weeks post ME in the monocular zones, starting from the borders of the visual cortex (Van Brussel et al. 2011; Nys et al. 2014). In this study, the same readout is used to investigate how activation of one interneuron-type affects the outcome of ME and whether it is correlated with the reactivation response following DE-pretreatment. Radioactive ISH experiments were performed on a series of coronal brain sections between bregma levels − 1.5 and − 5 mm and changes in the mRNA expression level of the immediate early gene (IEG) *zif268*, a proven excellent activity reporter gene in the mammalian brain, were quantified [mouse (van Brussel et al. 2009; Van Brussel et al. 2011; Woolley et al. 2013; Nys et al. 2014, 2015; Imbrosci et al. 2015; Smolders et al. 2015), cat (Arckens et al. 2000; Qu et al. 2003; Massie et al. 2003a, b; Leysen et al. 2004)]. As such, the spatial extent and the exact anatomical location of experience-induced, predominantly excitatory (Saffen et al. 1988; Cole et al. 1989; Worley et al. 1991; Chaudhuri et al. 1995; Kaczmarek and Chaudhuri 1997; Mataga et al. 2001), neuronal activity changes were analyzed and compared throughout all cortical layers of the visual and somatosensory neocortex of mice from all experimental conditions. ISH for *zif268*-mRNA was performed with a mouse-specific synthetic oligonucleotide probe (Eurogentec, Seraing, Belgium) with sequence 5′-ccgttgctcagcagcatcatctcctccagtttggggtagttgtcc-3′. As described previously (Arckens et al. 1995; Nys et al. 2015; Smolders et al. 2015), each probe was 3′-end labeled with {^33^P}dATP using terminal deoxynucleotidyl transferase (Invitrogen, Carlsbad, CA). Unincorporated nucleotides were separated from the labeled probe by means of miniQuick Spin Oligo Columns (Roche Diagnostics, Vilvoorde, Belgium). The cryostat sections were fixed, dehydrated and delipidated. The radioactively labeled probes were added to a hybridization cocktail [50% (v/v) formamide, 4× standard SSC buffer, 1× Denhardt’s solution, 10% (w/v) dextran sulfate, 100 µg/mL herring sperm DNA, 250 µg/mL tRNA, 60 mM dithiothreitol, 1% (w/v) *N*-lauroyl sarcosine, and 20 mM NaHPO_4_, pH 7.4] and applied to the cryostat sections (10^6^ cpm/section). After an overnight incubation at 37 °C in a humid chamber, the sections were rinsed in 1× standard SSC buffer at 42 °C, dehydrated, air-dried and exposed to an autoradiographic film (Biomax MR; Kodak, Rochester, NY). After 6 days, the films were developed in Kodak D19 developing solution and fixed in Rapid fixer (Ilford Hypam; Kodak). Autoradiographic images of adjacent sections per examined cortical area per mouse were scanned at 1200 dpi (CanoScan LIDE 600F; Canon, Tokyo, Japan), and all images were similarly adjusted for brightness and contrast in Adobe Photoshop CS5 (Adobe Systems, San Jose, CA). Pseudo-color maps were generated through a custom-made Matlab script (Matlab R2015b; Mathworks, Natick, MA) and represent a false color coding of the gray values; a low gray value is represented in black/green and a high gray value in white/yellow, indicating a low signal response or a high signal response, respectively, in accordance with a gray scale ranging from black (0) to white (255).

### Histology and localization of visual areal boundaries with Nissl patterns

Upon ISH, the cryostat sections were Nissl-counterstained (1% cresyl violet; Fluka, Sigma-Aldrich) according to standard procedures to visualize cortical boundaries between different visual and somatosensory areas and to aid the interpretation of the *zif268*-activity patterns. Images of the stained coronal sections were obtained at 5× (NA: 0.16) with a light microscope (Zeiss Axio Imager Z1) equipped with an AxioCam MRm camera (1388 × 1040 pixels) using the software program Zen (Zen Pro 2012, Carl Zeiss, Benelux). Immunofluorescent stainings for mCherry were performed on a series of post-fixed (4% PFA in PBS) cryosections adjacent to the Nissl-stained ISH sections to visualize the expression pattern of the SSFOs and compare it to the *zif268*-reactivation profile. This staining as well as the image acquisition were performed as explained in the above described section “Verifying the specificity and expression pattern of the viral vector transduction” using the Rabbit anti-RFP primary antibody, the Alexa Fluor 594 goat anti-rabbit IgG secondary antibody and the DAPI counterstaining.

Comparisons were made with the stereotaxic mouse brain atlas (Paxinos and Franklin 2012) to delineate visual and somatosensory cortical borders as described previously (van Brussel et al. 2009; Nys et al. 2015). In all figures illustrating visual cortex, large arrowheads indicate the total extent of the visual cortex, whereas small arrowheads indicate the interareal borders. Five subregions can be distinguished from medial to lateral: the medial extrastriate cortex (V2M), the primary visual cortex (V1) which is subdivided further into a monocular (V1m) and binocular (V1b) region, and the lateral extrastriate cortex (V2L), which is subdivided into binocular (V2Lb) and monocular (V2Lm) regions (Van der Gucht et al. 2007; van; Brussel et al. 2009; Nys et al. 2015). For somatosensory cortex, large arrowheads delineate the total extent of somatosensory cortex, the small arrowheads indicate interareal borders (Paxinos and Franklin 2012). Here, the primary barrel field cortex (S1BF) was used further in the analysis since the whisker input importantly contributes to cross-modal recovery within the visual cortex (Van Brussel et al. 2011).

### Quantitative analysis of ISH results

To quantify the optical density (OD; mean gray value per pixel) of the ISH autoradiograms, a custom-made Matlab (Matlab R2015b; Mathworks) script was used as described previously (Nys et al. 2015). For each condition, at least four mice were included and for each mouse, three consecutive ISH-sections between bregma levels − 3.0 and − 4.0 mm were loaded. Primary somatosensory cortex (S1) activity patterns were quantified on adjacent sections between − 1.82 and − 2.18 mm relative to bregma. The region of interest in the left hemisphere was demarcated by determining the top edge of the cortex, the boundary between the supra- and granular layers (II–III and IV), and the infragranular layers (V and VI) and the border between the infragranular layers and the corpus callosum. The region of interest was then divided equally into 24 segments from lateral to medial to create two lattices of 24 quadrangles, corresponding to the upper (II–IV) and lower (V–VI) layers. To compensate for possible variation in brain size and morphology, the lattices were translated on each autoradiogram over the cortical curvature, fixing the border of a specific segment to an areal border (border segment 19/20 is the area border V1m/V2M). For each segment created this way, the relative OD was calculated as the mean gray value of all pixels contained within a particular quadrangle and was normalized to the mean gray value of a square measured in the corpus callosum (a defined region with no *zif268*-expression above background) in order to compare autoradiograms across experiments. Relative neuronal activity was expressed in percentages based on the following formula: 1 − (cortical *zif268*/background) × 100.

For the top view images illustrating the *zif268*-mRNA levels over the entire extent of the visual cortex next to the SSFO-expression pattern, *zif268*-labeled sections every 100 µm between bregma levels − 2 and − 4 mm of 7wME and DE-Stim-7wME Cre-mice were provided with borders delineating V2L, V1, V2M and RM and were loaded into a custom made Matlab (Matlab R2015b; Mathworks) script as described in depth in Vreysen et al. (2017). Adjacent sections were stained for mCherry and by means of the DAPI counterstain these sections were superimposed onto the adjacent Nissl section, which was also matched with the *zif268*-expression pattern, creating an image with three channels (Photoshop CS6; Adobe Systems). As such both *zif268*- and SSFO-mCherry-stained sections could be imported into Matlab (Matlab R2015b; Mathworks). Again, the region of interest in the left hemisphere was demarcated by determining the top edge of the cortex, the boundary between the supra- and granular layers (II–III and IV), and the infragranular layers (V and VI) and the border between the infragranular layers and the corpus callosum. The region of interest between the rhinal fissure and the medial border of V2M was then divided equally into 50 segments from lateral to medial to create two lattices of 50 quadrangles, corresponding to the upper (II–IV) and lower (V–VI) layers. A small rectangular reference area was demarcated in the white matter to adjust for background signal. The mean optical density per segment and per channel was normalized by the reference region and then projected onto a horizontal planar projection. These horizontal projections from all slices of each animal were combined into a top view image.

### Statistics

For each condition, the data of relative OD-values in ISH-sections were presented as mean ± SEM. A normal distribution was verified and parallel equal variance between groups was tested. If a parametric test was allowed, a one-way ANOVA with Tukey post hoc test or an unpaired Student’s *t* test for pairwise comparison was used. For multiple factors, a two-way ANOVA was used to test for interactions. For all tests, a probability level (*α* level was set to 0.05) of < 0.05 was accepted as statistically significant (*P < 0.05, **P < 0.01, ***P < 0.001). Statistical analyses were performed using SPSS Statistics 24 software (IBM, Armonk, NY).

## Results

We optogenetically stimulated SST-interneurons within the visual cortex on 3 consecutive days, twice per day, to investigate if local induction of SST-interneuron activity prior to, or 3 weeks after, monocular enucleation (ME) would affect the ensuing cortical reactivation of binocular and/or monocular cortical territory. To judge the cortical reactivation process, we quantitatively measured *zif268*-mRNA expression, throughout the visual cortex and compared it to that of non-stimulated ME-mice and mice that underwent a DE-pretreatment. This allowed us to investigate whether unimodal open-eye-driven cortical plasticity in the binocular region and whisker-driven cross-modal plasticity in medial monocular cortex were affected because of the SST-interneuron activation as pre- and/or post-treatment strategies. Figure 1 illustrates the experimental timeline of all studied conditions as well as relevant control conditions.

### Validation of cell-type specific SSFO-expression, and functionality in SST-interneurons in adult mouse visual cortex

**Fig. 2:**
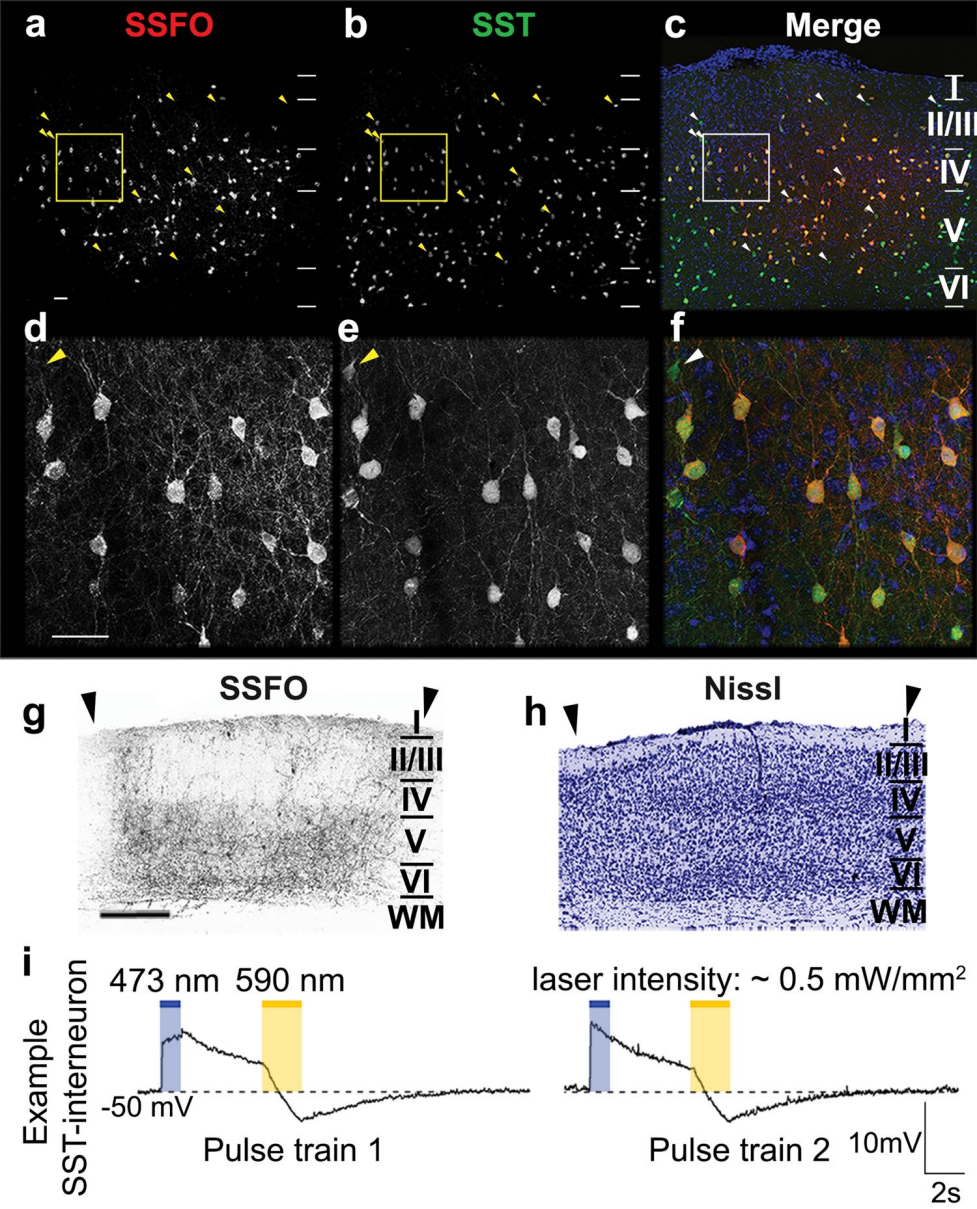
Expression pattern and functionality of SSFOs in SST-interneurons following viral vector transduction. Injection site in V1 on a coronal section illustrating SSFO-transduced neurons (**a**), eYFP-positive SST-interneurons (**b**) and an overlap between SSFO-positive neurons in red, eYFP-positive SST-interneurons in green and a DAPI counterstain in blue (**c**). Horizontal lines demarcate the laminar borders within the neocortex. Higher magnification details from the yellow and white rectangular regions in **a**–**c**. SSFO-positive neurons (**d**) and eYFP-positive SST-interneurons (**e**) are overlapped in red and green, respectively, with a blue DAPI counterstain (**f**). Yellow or white arrowheads indicate eYFP-positive SST-interneurons that do not express SSFO. Scale bars = 100 µm. Detail of the laminar SSFO-expression pattern at bregma level − 3.40 mm of a Stim-7wME animal (**g**) and its adjacent Nissl-stained cryosection (**h**) illustrate normal histology despite long term implantation of the head posts. Arrowheads delineate V1. Scale bar = 500 µm. **i** Example recordings from a SST-interneuron illustrate the responses of its membrane potential following light stimulation. A 1-s blue (473 nm) light pulse (intensity ~ 0.5 mW/mm^2^) increased the membrane potential to − 40 V, which then gradually decayed. A subsequent yellow (590 nm) light pulse (intensity ~ 0.5 mW/mm^2^) allowed the cell to return to the prestimulation baseline level as SSFO-channels close. Two consecutive stimulations within the same SST-interneuron are shown

To investigate the role of SST-interneurons in visual cortex plasticity using optogenetics, we specifically directed the expression of stable-step function opsins (SSFOs) towards this interneuron population within the visual cortex. We confirmed cell-specificity of the Cre-dependent viral vector transduction (rAAV2/7-CMV-flex-SSFO-mCherry) in SST-eYFP-Cre mice by means of immunofluorescent double-stainings for eYFP-expressing SST-interneurons and mCherry-tagged SSFOs (Fig. 2a–f). Within the transduction site of the vector, the majority of SST-positive interneurons co-express SSFOs at the plasma membrane. Arrowheads indicate a minority of SST-positive cell bodies within the injection site where no detectable levels of SSFO can be discerned. Although all cortical layers (except layer I) show SSFO-transduced SST-somata, the expression is most prominent in layers IV–VI (Fig. 2a). This is further illustrated in Fig. 2g, where an SSFO-mCherry-stained visual cortex cryosection (bregma − 3.40 mm) of a Stim-7wME mouse reveals the majority of SSFO-positive neurons and neurites present in infragranular layers V and VI within V1, in addition to SSFO-positive neurites in layer I. The latter corresponds to the morphology of Martinotti cells, abundantly sending out axon collaterals towards layer I (Wang et al. 2004). The Nissl staining on an adjacent cryosection (Fig. 2h) reveals a normal morphological pattern across all neocortical layers and an intact layer I, despite the long-term presence of the optic fiber implant at this location.

To verify the functionality of the viral vector construct and to check whether SSFOs can be activated and deactivated reproducibly by blue and yellow light stimulation respectively (Yizhar et al. 2011), we performed whole-cell patch clamp recordings in current clamp mode of SSFO-positive SST-interneurons in combination with blue (473 nm) or yellow (590 nm) light pulses. Activation of SSFOs by a 1-s 473 nm blue light pulse depolarizes the SSFO-expressing SST-interneuron (Fig. 2i, blue stimulation epoch), thereby rendering the neuron more excitable and responsive to endogenous activity (Yizhar et al. 2011; Tye and Deisseroth 2012). The SSFO-activation can be successfully reversed by a 2-s 590 nm light pulse, which in turn causes an instantaneous repolarization of the membrane to pre-stimulation baseline levels (Fig. 2i, yellow stimulation epoch).

### *Zif268* expression in visual cortex is not affected by optic fiber implantation

**Fig. 3:**
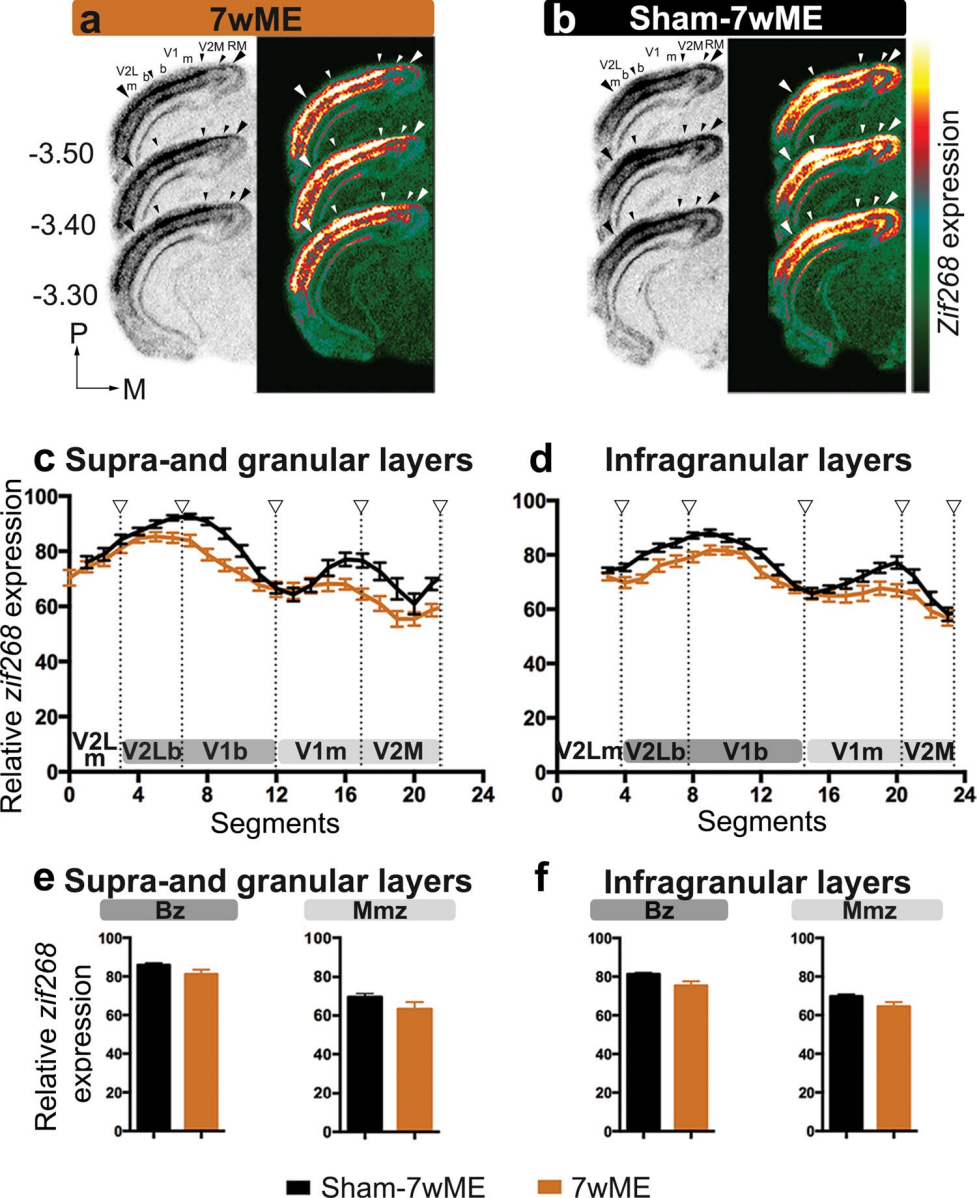
The effect of sham surgery on the recovery pattern following 7wME in the deprived visual cortex. Images of three adjacent *zif268*-mRNA labeled coronal cryosections surrounding bregma level − 3.40 mm of normal 7wME mice (**a**) and sham-7wME mice (**b**) show a similar reactivation profile. The corresponding pseudocolor representations of signal intensity differences are displayed next to their respective ISH sections. Scale bar = 2 mm. Line graphs illustrating the relative *zif268*-mRNA expression level measured as the average OD-value per segment for sham-7wME mice (black line) and normal 7wME mice (orange line). For supra- and granular layers II/III and IV (**c**) and infragranular layers V and VI (**d**) the expression levels are displayed along the five predefined visual subdivisions (hollow arrowheads are in accordance with the small arrowheads in **a** and **b**, including the subdivision between monocular zones m and binocular zones b). Error bars represent the SEM of the mean OD in each segment. Relative *zif268*-mRNA expression levels are shown as OD-values averaged over the binocular zone Bz and the medial monocular zone Mmz for supra- and granular layers (**e**) and infragranular layers (**f**). Bz corresponds to V2Lb combined with V1b (dark grey marking), Mmz corresponds to V1m combined with V2M (light grey marking) as illustrated in **c** and **d**. Error bars are SEM. Comparison of black bar graphs representing the OD-values of sham-7wME mice to orange bar graphs of normal 7wME mice indicated no differences between the two conditions in either layer or zone (sham-7wME, upper layers, Bz: average ± SEM; 85.87 ± 0.99; Mmz: 69.48 ± 1.90; lower layers, Bz: 81.29 ± 0.76; Mmz: 69.72 ± 1.09; 7wME, upper layers, Bz: 81.16 ± 2.34; Mmz: 63.53 ± 3.47; lower layers, Bz: 75.26 ± 2.34; Mmz: 64.47 ± 2.34)

To rule out that the presence of the optic fiber implant or the blue light pulse regime per se hampers ME-induced cortical reactivation, we compared the level of *zif268* expression of the visual cortex between 7wME and Sham-7wME. The latter expressed an eGFP-fluorophore instead of the light-activatable SSFO, but underwent the same light-stimulation protocol (Figs. 1, 3). Background corrected OD-values of the experience-induced *zif268* expression around bregma level − 3.40 mm (Fig. 3a, b) [where generally a strong influence on the recovery pattern following ME can be observed (Van Brussel et al. 2011)] reach similar levels in 7wME and Sham-7wME mice (Fig. 3c, d), indicating that the presence of the head implant, or the blue laser pulses per se, do not influence the cortical recovery potential. No difference between these conditions is observed in supra- and granular layers (Fig. 3c), or in infragranular layers (Fig. 3d). In each visual subregion along the lateromedial extent of the visual cortex (monocular extrastriate lateral visual cortex V2Lm, V1m, V1b, and V2M), *zif268* expression levels are similar between 7wME and Sham-7wME (Fig. 3c, d) (independent samples *t* test: V2Lm upper layers: *P* = 0.353; lower layers: *P* = 0.111; V1b upper layers: *P* = 0.325; lower layers: *P* = 0.120; V1m upper layers: *P* = 0.480; lower layers: *P* = 0.409; V2M upper layers: *P* = 0.131; lower layers: *P* = 0.065; Fig. 3c, d). Only V2Lb in sham-7wME reveals slightly higher *zif268* levels (upper layers, *P* = 0.039; lower layers, *P* = 0.048).

As the recovery of the binocular and monocular zone differs in nature, relying more on open eye potentiation or cross-modal recruitment respectively (Van Brussel et al. 2011), we assessed the reactivation profiles separately for the binocular (binocular V2L and primary visual cortex V1) and the medial monocular (monocular V1 and V2M) zones, further referred to as Bz and Mmz (Fig. 3e, f). Comparison between 7wME and sham-7wME reveals no differences in *zif268* expression for either the Bz or Mmz (independent samples *t* test: supra- and granular layers Bz: *P* = 0.124; Mmz: *P* = 0.232; infragranular layers Bz: *P* = 0.061; Mmz: *P* = 0.105). Overall, these reactivation profiles are similar to what has been observed in previous studies in 7wME C57Bl/6J mice (Nys et al. 2014). Based on the similarity of these reactivation profiles, Sham-7wME served as control for comparison with all other optogenetic stimulation conditions.

### Short-term stimulation of SST-interneuron activity prior to ME induction impedes the reactivation of the visual cortex

**Fig. 4:**
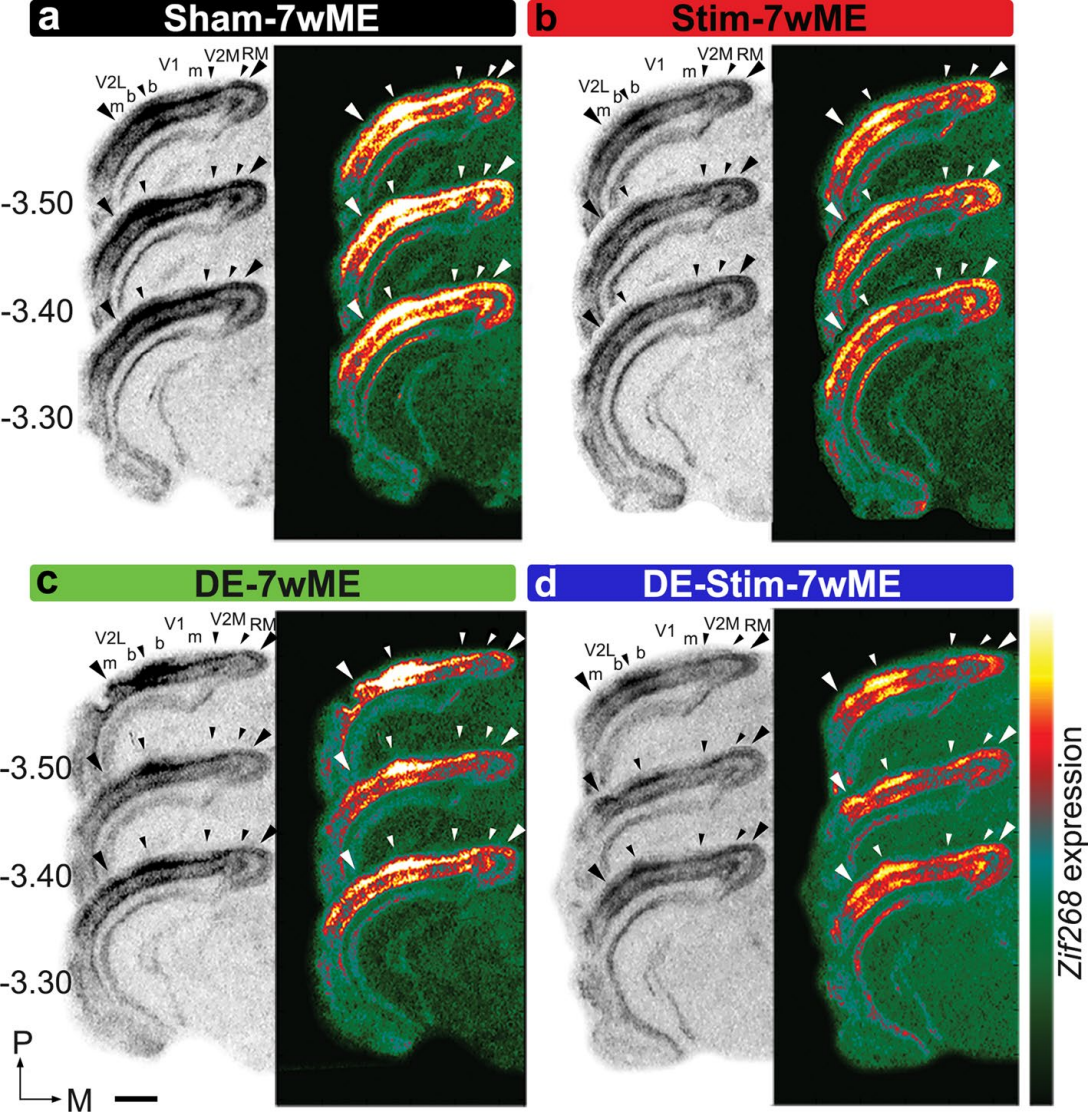
Effects of optogenetic SST-interneuron activation and/or DE-pretreatment on the recovery profile of the deprived visual cortex after 7wME. Images of three adjacent *zif268*-mRNA labeled coronal cryosections surrounding bregma level − 3.40 mm of a sham-7wME mouse (**a**), a Stim-7wME mouse (**b**), a DE-7wME mouse (**c**) and a DE-Stim-7wME mouse (**d**). Both stimulation (**b**) and DE (**c**) as well as the combination of both manipulations (**d**) result in a decreased reactivation profile of the deprived visual cortex following 7wME compared to the sham-7wME mouse (**a**). The corresponding pseudocolor representations of signal intensity differences are displayed next to their respective ISH sections. Scale bar = 2 mm, *P* posterior, *M* medial

To study if short-term activation of SST-interneurons at the time of ME-onset affects the reactivation of the deprived visual cortex after 7wME, we optogenetically stimulated SST-interneurons in V1 contralateral to ME (Stim-7wME, Fig. 1) in awake, freely-moving mice in a standard day-time light environment. The significantly lower *zif268* expression levels in the ME-deprived visual cortex of Stim-7wME compared to Sham-7wME controls expose the reduced capacity for reactivation in supra- and granular, and infragranular layers in the weeks following the SST-interneuron activation (Fig. 4a, b).

The low reactivation is reminiscent of the previously revealed effect of a 10-day dark exposure (DE)-treatment prior to ME on the long-term plastic response following vision loss. Indeed, Nys et al. (2015) demonstrated that the cross-modal component of recovery is not, or only weakly recruited when mice are kept in the dark prior to 7wME, which was observed as a low reactivation of the Mmz. In the present study, activating SST-interneurons prior to ME results in a response comparable to DE (Fig. 4b, c). Interestingly, a combinatorial pretreatment strategy consisting of SST-interneuron stimulation during DE prior to ME (DE-Stim-7wME) results in strongly reduced *zif268* levels in both supra- and granular and infragranular layers compared to either pretreatment strategy separately, indicating an additive effect of DE and SST-interneuron stimulation in blocking the long-term cortical reactivation process (Fig. 4d).

**Fig. 5:**
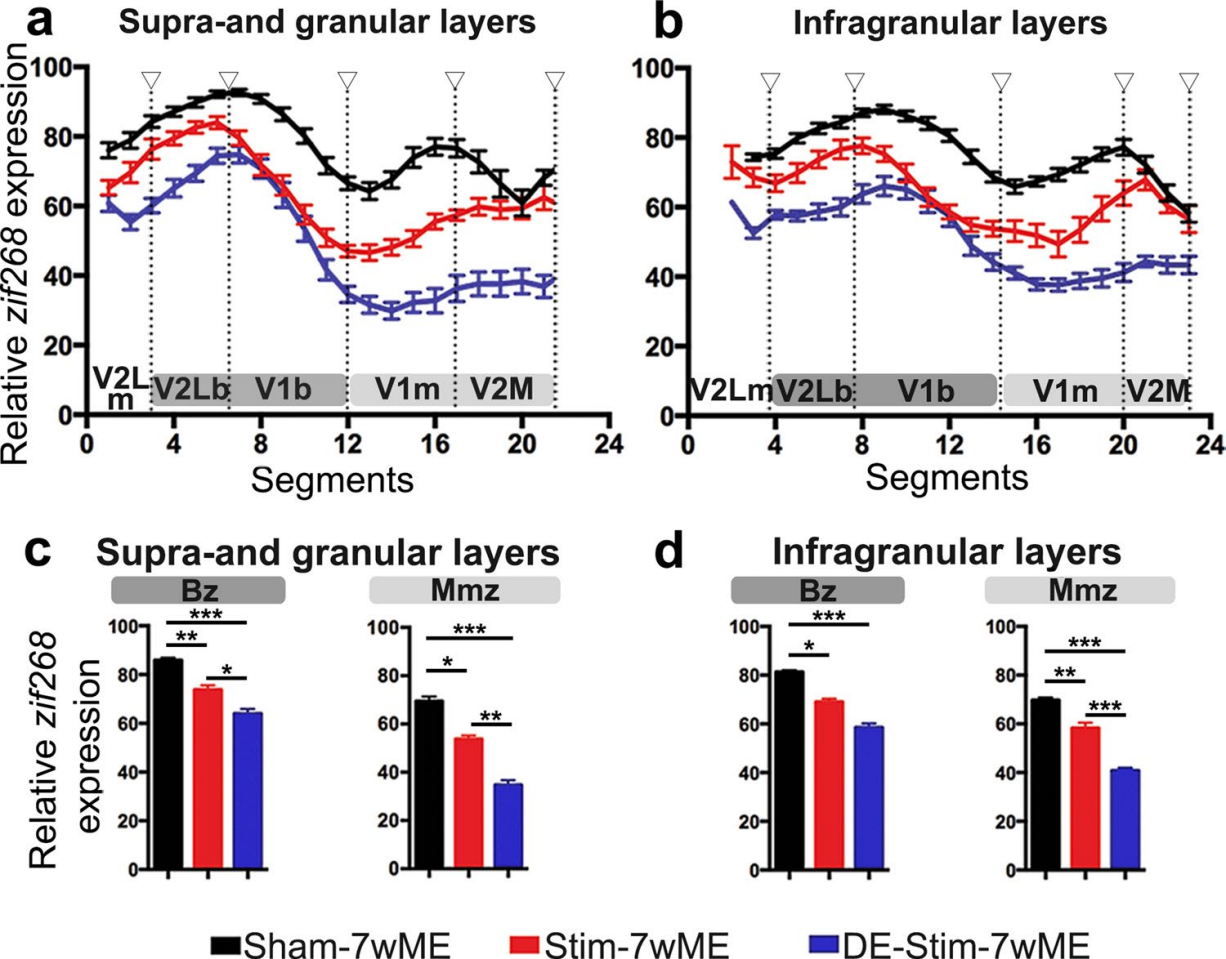
Comparison of the reactivation profile of the deprived visual cortex in Stim-7wME (red) and DE-Stim-7wME mice (blue) versus Sham-7wME mice (black). Line graphs illustrate the relative *zif268*-mRNA expression level measured as the average OD-value per segment (0–24) from lateral to medial for sham-7wME mice, Stim-7wME mice and DE-Stim-7wME mice. The expression levels are displayed in relation to the visual subdivisions separated by hollow arrowheads which are in accordance with the small arrowheads in Fig. 4 a–d, including the subdivision between monocular zones m and binocular zones b, separately for supra- and granular layers II/III and IV (**a**) and infragranular layers V and VI (**b**). Error bars represent SEM. Relative *zif268*-mRNA expression levels are displayed as OD-values averaged over the binocular zone, Bz and the medial monocular zone, Mmz for upper (**c**) and lower layers (**d**). Bz corresponds to V2Lb combined with V1b (dark grey) and Mmz to V1m and V2M (light grey) as displayed in **a, b**. Error bars are SEM. Comparison of the OD-values of sham-7wME mice and Stim-7wME mice to DE-Stim-7wME mice indicates severely lowered reactivation levels across all cortical zones and layers for stimulated and DE-Stim animals (sham-7wME, upper layers, Bz: Average ± SEM; 85.87 ± 0.99; Mmz: 69.48 ± 1.90; lower layers, Bz: 81.29 ± 0.76; Mmz: 69.72 ± 1.09; Stim-7wME, upper layers, Bz: 73.83 ± 1.71; Mmz: 63.99 ± 1.91; lower layers, Bz: 69.00 ± 1.37; Mmz: 58.33 ± 2.17; DE-Stim-7wME, upper layers, Bz: 63.99 ± 1.91 ; Mmz: 34.72 ± 1.90; lower layers, Bz: 58.57 ± 1.61; Mmz: 40.78 ± 1.14). *P ≤ 0.05, **P ≤ 0.001, ***P ≤ 0.0001

Quantification of these reactivation profiles as normalized OD-values of *zif268* expression along the lateromedial extent of the ME-affected visual cortex, indeed confirms the significantly different levels of reactivation. Highest *zif268* levels were present in Sham-7wME controls, reduced *zif268* levels in SST-interneuron stimulated 7wME mice, and the strongest reduction in *zif268* expression was observed when SST-interneurons were activated during the DE-pretreatment, in upper (Fig. 5a) as well as in lower layers (Fig. 5b). The pretreatment strategy before ME (SST-interneuron stimulation, SST-interneuron stimulation during DE, or no manipulation) has a significant effect on *zif268* expression (One-way ANOVA, upper layers, Bz: *P* < 0.001; Mmz: *P* < 0.001; lower layers, Bz: *P* = 0.001; Mmz: *P* < 0.001). Multiple comparisons *post hoc* tests confirmed that *zif268* expression both in Bz and in the Mmz are affected, but especially in the Mmz SST-interneuron stimulation during DE results in the strongest decrease in recovery (Sham-7wME versus Stim-7wME, upper layers, Bz: *P* = 0.007; Mmz: *P* = 0.017; lower layers, Bz: *P* = 0.046; Mmz: *P* = 0.006; Stim-7wME versus DE-Stim-7wME, upper layers, Bz: *P* = 0.016; Mmz: *P* = 0.003; lower layers, Bz: *P* = 0.074; Mmz: *P* < 0.001; Sham-7wME versus DE-Stim-7wME, upper layers, Bz: *P* < 0.001; Mmz: *P* < 0.001; lower layers, Bz: *P* = 0.001; Mmz: *P* < 0.001; Fig. 5c, d).

Considering that the expression of SSFOs was limited to V1 and did not spread into extrastriate V2M or V2L, we further examined these visual subdivisions constituting the Bz and Mmz. A significant effect of the pretreatment strategy on the *zif268*-mRNA expression profile was found in all visual subdivisions, in upper and lower layers (One-way ANOVA, upper layers, V2Lm: *P* = 0.001; V2Lb: *P* < 0.001; V1b: *P* < 0.001; V1m: *P* < 0.001; V2M: *P* = 0.001; lower layers, V2Lm: *P* = 0.010; V2Lb: *P* = 0.001; V1b: *P* = 0.001; V1m: *P* < 0.001; V2M: *P* < 0.001, Fig. 5a, b).

*V2M* In extrastriate V2M, the *zif268* expression levels are similar in Stim-7wME and Sham-7wME controls (multiple comparisons *post hoc*: upper layers: *P* = 0.286; lower layers: *P* = 0.081), but the addition of DE to the SST-interneuron activation results in highly reduced *zif268* levels compared to Stim-7wME and Sham-7wME controls (DE-Stim-7wME versus Sham-7wME: upper layers: *P* = 0.001; lower layers: *P* < 0.001; DE-Stim-7wME versus Stim-7wME: upper layers: *P* = 0.013; lower layers: *P* < 0.001).

*V2L* In lateral extrastriate regions V2Lb and V2Lm, *zif268* levels are also lower in DE-Stim-7wME compared to Sham-7wME controls (upper layers, V2Lm: *P* = 0.001; V2Lb: *P* < 0.001; lower layers, V2Lm: *P* = 0.008; V2Lb: *P* = 0.001). SST-interneuron stimulation without DE results in only mildly lower *zif268* levels compared to Sham-7wME controls, which is only significant in upper, but not in lower layers (upper layers, V2Lm: *P* = 0.021; V2Lb: *P* = 0.019; lower layers, V2Lm: *P* = 0.171; V2Lb: *P* = 0.086) (Fig. 5a, b).

*V1* In V1, *zif268* levels are lower in Stim- as well as DE-Stim-7wME compared to Sham-7wME controls (Stim-7wME versus Sham-7wME: upper layers, V1m: *P* = 0.001; V1b: *P* = 0.011; lower layers, V1m: *P* = 0.007; V1b: *P* = 0.037; DE-Stim versus Sham-7wME: upper layers, V1m: *P* < 0.001; V1b: *P* < 0.001; lower layers, V1m: *P* < 0.001; V1b: *P* = 0.001). The addition of DE again results in a significantly lower reactivation in V1m (Stim-7wME versus DE-Stim-7wME, upper layers: *P* < 0.001, lower layers: *P* = 0.010). In the binocular region V1b, *zif268* levels are also lower in DE-Stim-7wME compared to Stim-7wME in upper, but not in lower layers (upper layers: *P* = 0.031; lower layers: *P* = 0.133) (Fig. 5a, b).

In summary, the strongest effect on reactivation was seen when SST-interneurons were stimulated prior to ME during DE. The lack of reactivation following Stim-7wME appears more strongly confined to primary visual cortex (V1b and V1m), the actual region of SSFO transduction, whereas in combination with DE, recovery following ME is impeded across the entire mediolateral extent of the visual cortex, specifically spreading out into extrastriate area V2L and V2M.

### Combined DE and SST-interneuron stimulation prior to ME acts as a strong blockade preventing reactivation over the entire extent of the visual cortex

**Fig. 6:**
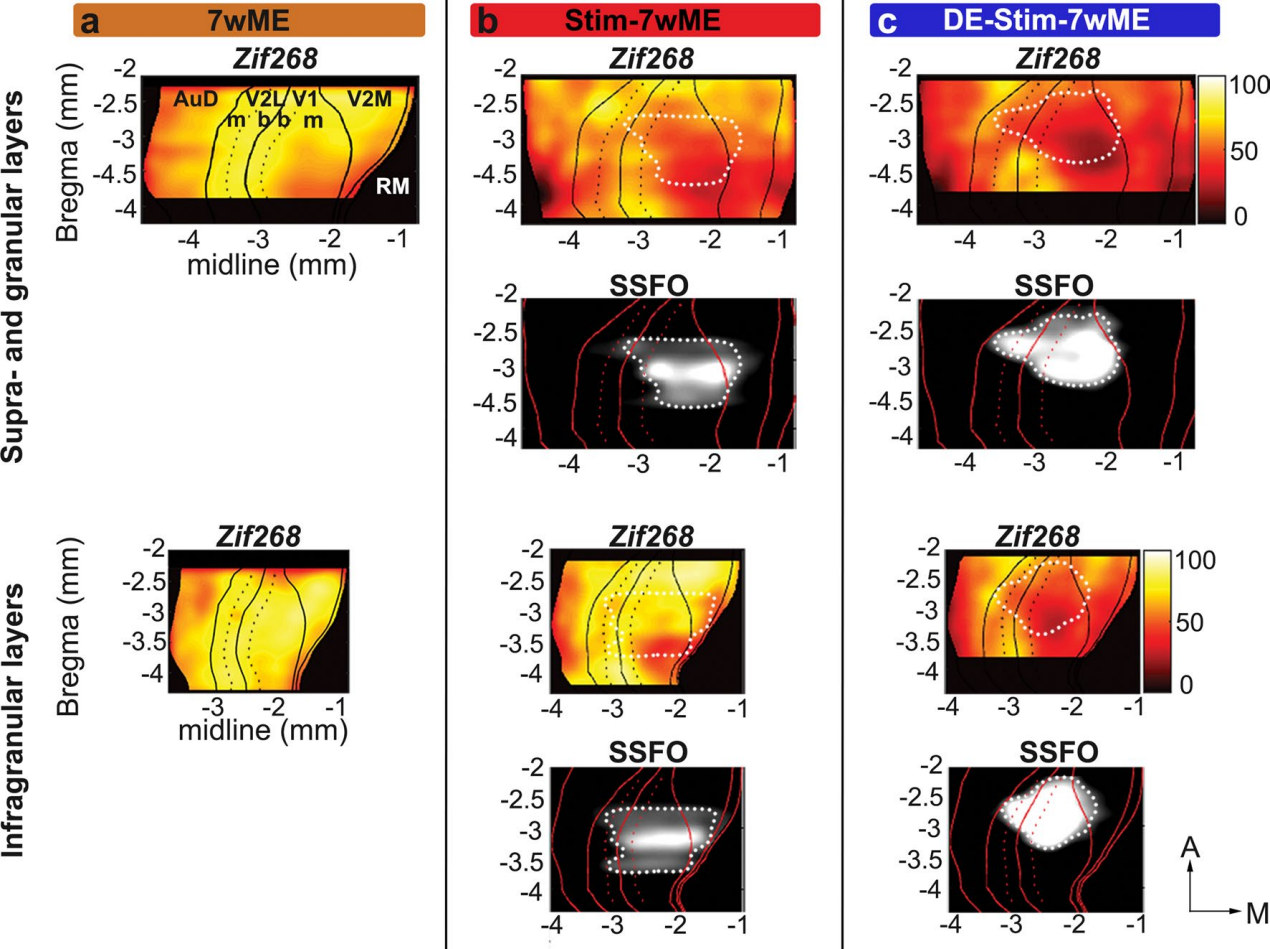
Localized SSFO-stimulation results in a lowered reactivation profile specifically at and posterior of the stimulation site, whereas reactivation in DE-Stim-7wME mice spreads out beyond the transduced cortical region into extrastriate regions over the entire extent of the visual cortex. Top view heat map representations of OD-values of *zif268*-mRNA expression and SSFO-expression per slice and segment in upper (top panels) and lower layers (bottom panels) across the entire extent of the visual cortex. Black lines in *zif268-*top views and red lines in the SSFO-top views depict the borders between auditory cortex (AuD), V2L, V1, V2M and rostromedial area (RM), black and red dotted lines separate monocular, m, from binocular, b, regions in V2L and V1. Top view representation of a 7wME mouse (**a**) visualizes complete recovery across the visual cortex, except for a small region medial and posterior in supragranular layers. Top view representation of a representative Stim-7wmE mouse (**b**) visualizes a lowered reactivation profile specifically at the posteromedial edges of the injection site. Top view representation of a representative DE-Stim-7wME mouse (**c**) visualizes the lack of recovery after 7wME, not only at the site of SSFO-expression, but throughout the entire visual cortex with the most pronounced drop in reactivation posterior and medial of the SSFO-expression site. *X*- and *Y*-axes represent midline and bregma levels, respectively in mm. White dotted lines delineate the injection site on the lower black and white SSFO-expression panels and are projected onto the upper *zif268* expression panels for interpretation of the *zif268* expression data. *A* anterior, *M* medial

To assess the full anteroposterior and mediolateral extent of the ongoing decreased cortical reactivation in de weeks following the optogenetic SST-interneuron stimulation, and to line-up the data with the spatial extent of the SSFO expression, we generated top view heat map representations (Vreysen et al. 2017) illustrating the cortical *zif268* expression levels as well as the SSFO-expression site (dotted white lines) between bregma levels − 2.20 and − 4 mm (Fig. 6).

A first example case of a top view from a non-stimulated 7wME control (Fig. 6a) illustrates the expected strong reactivation profile for supra- and granular and infragranular layers over almost the entire visual cortex, encompassing binocular and monocular cortical territory. The heat map representation confirms that only at the most posterior, medial cortical coordinates *zif268* expression remains lower. This is in accordance with previous reports (Van Brussel et al. 2011; Nys et al. 2014; Vreysen et al. 2017) which showed that apart from open eye potentiation of mainly the binocular zone, whisker inputs invade the visual cortex from regions located nearest to somatosensory cortex towards more posterior locations, in an anteroposterior fashion (Van Brussel et al. 2011). The recovery is strongest in lower cortical layers, in accordance with the stronger cross-modal drive previously observed in infragranular layers (Van Brussel et al. 2011).

A heat map representation of the Stim-7wME condition (Fig. 6b) illustrates the reduced cortical reactivation compared to 7wME controls (Fig. 6a) in upper and lower layers. Especially at posterior, medial edges of the SSFO-expression site, *zif268* expression was highly reduced compared to the 7wME controls. Further posteromedial of the injection site, *zif268*-levels remain low, suggesting that the local SST-interneuron modulation blocks the reactivation spreading out towards posterior edges of the ME-affected visual cortex. In the binocular region (demarcated by the black dashed lines), lateral to the SSFO expression site and at the border between V2L and V1, ipsilateral spared-eye inputs remain and *zif268* expression levels are comparable to 7wME controls, especially in infragranular layers, indicating that cross-modal takeover in Mmz is hampered more strongly.

The DE-Stim-7wME visual cortex heat map top view (Fig. 6c) illustrates a poorly reactivated visual cortex, in upper as well as lower layers, including extrastriate medial, and to a lesser extent the lateral areas. In contrast with Stim-7wME animals, the lowered reactivation is not limited to regions posterior to the injection site. Rather, cortical regions anterior of V2M, where reactivation starts and gradually invades more posterior regions of the visual cortex (Vreysen et al. 2017), also lack the reactivation seen in 7wME or Stim-7wME animals. Basically, activity is still present in binocular regions, albeit also lower compared to 7wME or Stim-7wME animals at more anterior coordinates.

In summary, the different levels of reactivation, gradually decreasing from 7wME controls, to Stim-7wME to DE-Stim-7wME are visible over the entire extent of the visual cortex. When SST-interneurons are stimulated during DE prior to ME, reactivation of the visual cortex is maximally impeded from posterior up to anterior extrastriate regions.

### SST-interneuron stimulation does not affect the response of non-visual cortical areas to long-term ME, in contrast to DE-pretreatment

**Fig. 7:**
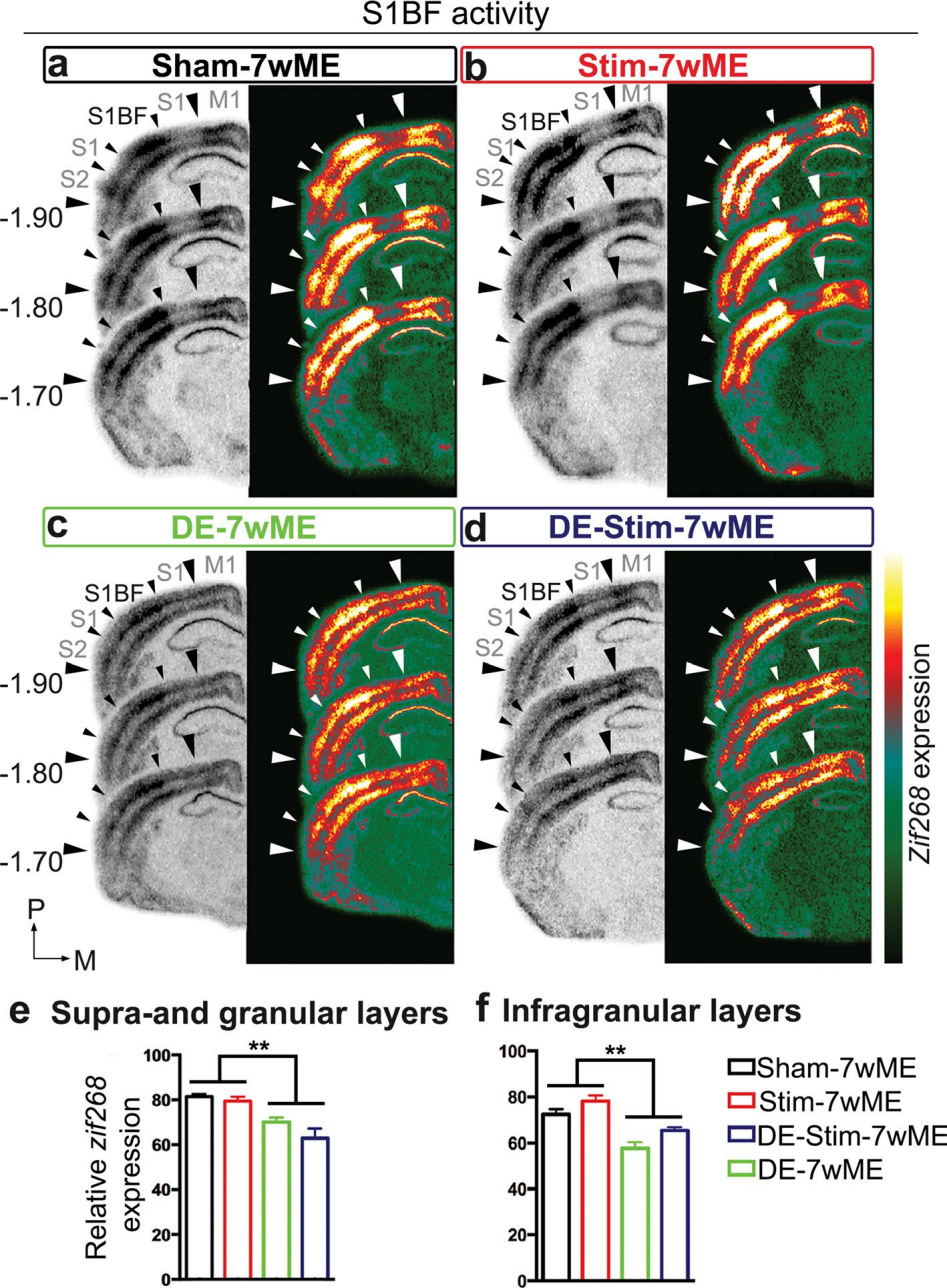
DE-pretreatment affects S1BF activity after 7wME. Images of three adjacent *zif268*-mRNA labeled coronal cryosections surrounding bregma level − 1.80 mm depicting the somatosensory cortex of a sham-7wME mouse (**a**), a Stim-7wME mouse (**b**), a DE-7wME mouse (**c**) and a DE-Stim-7wME mouse (**d**). DE-pretreatment (**c, d**) resulted in lower *zif268*-levels in S1BF compared to non-DE-pretreated animals (**a, b**). The corresponding pseudocolor representations of signal intensity differences are displayed next to their respective ISH sections. Scale bar = 2 mm, *P* posterior, *M* medial. Relative *zif268*-mRNA expression levels are displayed as OD-values averaged over S1BF for upper (**e**) and lower layers (**f**). Error bars are SEM. DE-pretreated animals showed a significantly lower activity level in S1BF in both upper and lower layers compared to non-DE-pretreated animals, regardless of whether they were optogenetically stimulated or not [sham-7wME (*n* = 4), upper layers: average ± SEM; 81.54 ± 1.30; lower layers: 72.47 ± 2.55; DE-7wME (*n* = 5), upper layers: 70.18 ± 1.97; lower layers: 57.59 ± 2.78; Stim-7wME (*n* = 3), upper layers: 79.54 ± 1.82; lower layers: 78.11 ± 2.54; DE-Stim-7wME (*n* = 4), upper layers: 62.92 ± 4.37; lower layers: 65.40 ± 1.48]. **P ≤ 0.001

Previous work has shown that upon ME, the intact sensory modalities adjacent to the deprived visual cortex become more strongly activated, in particular when visual cortex reactivation encompasses cross-modal plasticity. Furthermore, when the cross-modal component of reactivation is less recruited, as is the case when mice are dark exposed prior to 7wME, non-visual areas show significantly lower activity levels compared to those areas in normally sighted age-match controls (Nys et al. 2014, 2015). To verify whether increased SST-interneuron activity within V1 affects adjacent intact areas in how they are recruited to establish cross-modal take-over, we compared activity levels in the somatosensory barrel field, S1BF, between DE versus non-DE and SST-interneuron stimulated versus non-stimulated sham conditions (Fig. 7a–d). *Zif268* levels in S1BF surrounding bregma level − 1.80 mm in Sham-7wME controls and Stim-7wME conditions are clearly higher compared to DE-7wME and DE-Stim-7wME conditions. A two-way ANOVA analysis reveals no interaction between the effects of DE or SST-interneuron stimulation (upper layers: *P* = 0.655; lower layers: *P* = 0.343). Instead, activity levels are only affected by DE-pretreatment (upper layers: *P* = 0.001; lower layers: *P* = 0.001) and not by the presence or absence of SST-interneuron stimulation (upper layers: *P* = 0.052; lower layers: *P* = 0.075). An independent samples *t* test between both non-DE conditions (Sham-7wME and Stim-7wME) and DE-conditions (DE-7wME and DE-Stim-7wME) indeed indicates that non-DE conditions show significantly higher activity levels in S1BF compared to DE conditions (upper layers: *P* = 0.001; lower layers: *P* = 0.001) (Fig. 7e, f).

In summary, these results indicate that increased barrel cortex activity due to ME cannot overrule the effect of local SST-interneuron prestimulation in V1. When SST-interneuron stimulation is combined with the more brain-wide DE-induced effects, including those on the intact sensory modalities, the reactivation of the deprived cortical modality is suppressed much stronger compared to what either treatment can elicit on its own (Fig. 4b–d).

### Late onset SST-interneuron stimulation with or without DE-pretreatment has a milder impact on cortical recovery

**Fig. 8:**
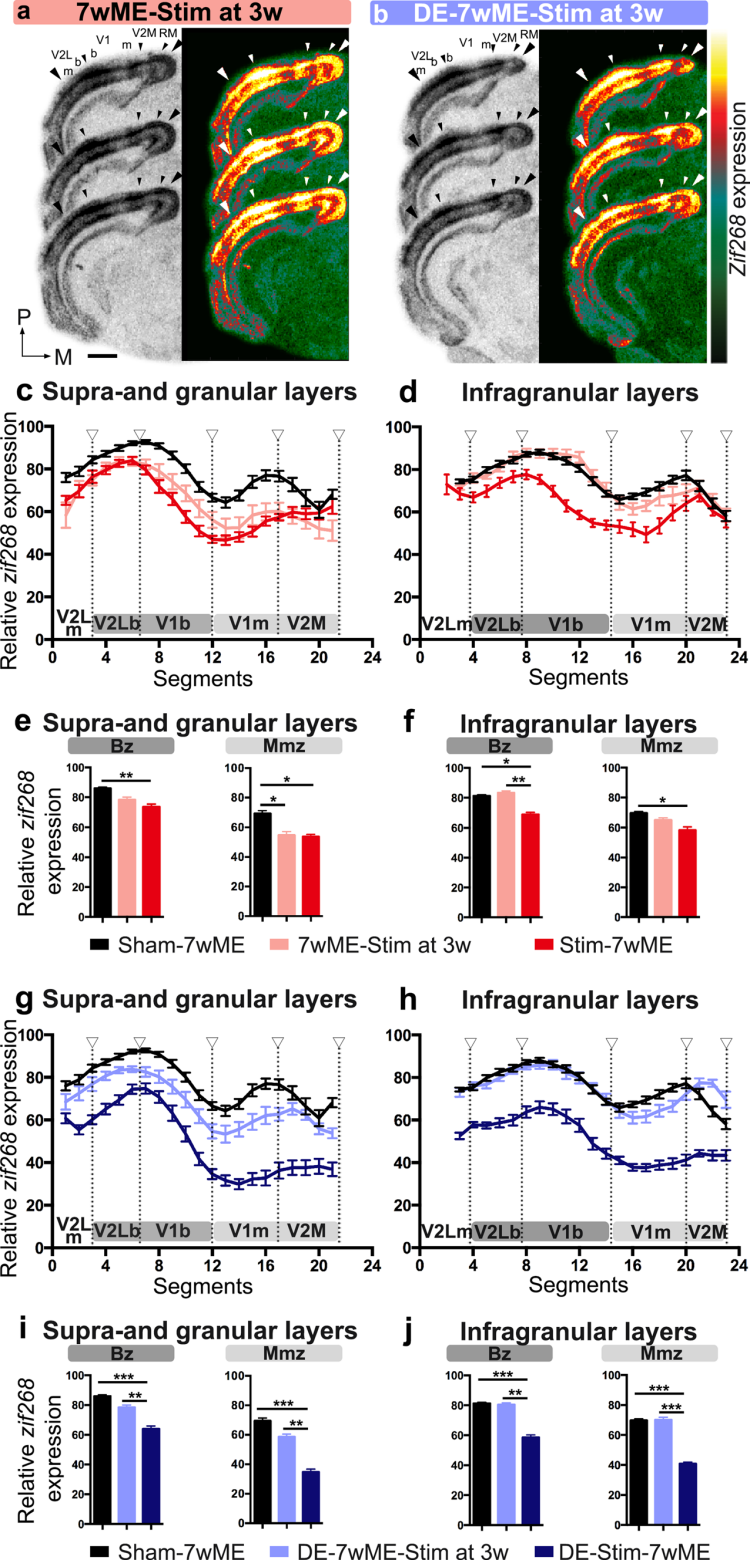
Comparison of the reactivation profile of the deprived visual cortex in pre-stimulated [Stim-7wME (red), DE-Stim-7wME mice (blue)] versus late stimulated mice [7wME-Stim at 3w (pink) and DE-7wME-Stim at 3w (light blue)], and Sham-7wME mice (black). Images of three adjacent *zif268*-mRNA labeled coronal cryosections surrounding bregma level − 3.40 mm of a 7wME-Stim at 3 weeks post-ME (3w) mouse (**a**), and a DE-7wME-Stim at 3w mouse (**b**). The resulting activity pattern was less strongly downregulated compared to the Sham-7wME mouse (Fig. 4a). The corresponding pseudocolor representations of signal intensity differences are displayed next to their respective ISH sections. Scale bar = 2 mm, *P* posterior, *M* medial. Line graphs illustrate the relative *zif268*-mRNA expression level measured as the average OD-value per segment (0–24) from lateral to medial for sham-7wME mice, 7wME-Stim at 3w and Stim-7wME mice (**c, d**), and sham-7wME mice, DE-7wME-Stim at 3w and DE-Stim-7wME mice (**g, h**). The expression levels are displayed in relation to the visual subdivisions separated by hollow arrowheads which are in accordance with the small arrowheads in **a, b**, including the subdivision between monocular zones m and binocular zones b, separately for supra- and granular layers II/III and IV (**a**) and infragranular layers V and VI (**b**). Error bars represent SEM. Relative *zif268*-mRNA expression levels are displayed as OD-values averaged over the binocular zone, Bz and the medial monocular zone, Mmz for upper (**e, i**) and lower layers (**f, j**). Bz corresponds to V2Lb combined with V1b (dark grey) and Mmz to V1m and V2M (light grey) as displayed in **c, d, g, h**. Error bars are SEM. Comparison of the OD-values of sham-7wME mice, 7wME-Stim at 3w and Stim-7wME mice, reveal that stimulation after 3wME results in a layer-specific intermediate activity pattern, in infragranular layers the activity pattern was more similar compared to sham-7wME and higher compared to Stim-7wME, in supra- and granular layers the activity pattern was lower compared to sham-7wME and similar to Stim-7wME. In DE-conditions, the activity pattern of DE-7wME-Stim at 3w mice was similar to sham-7wME, but higher compared to DE-Stim-7wME in supra- and granular, and infragranular layers (sham-7wME, upper layers, Bz: average ± SEM; 85.87 ± 0.99; Mmz: 69.48 ± 1.90; lower layers, Bz: 81.29 ± 0.76; Mmz: 69.72 ± 1.09; 7wME-Stim at 3w, upper layers, Bz: 78.32 ± 1.77; Mmz: 54.77 ± 2.44; lower layers, Bz: 83.32 ± 1.25; Mmz: 65.01 ± 1.50; Stim-7wME, upper layers, Bz: 73.83 ± 1.71; Mmz: 63.99 ± 1.91; lower layers, Bz: 69.00 ± 1.37; Mmz: 58.33 ± 2.17; DE-7wME-Stim at 3w, upper layers, Bz: 78.36 ± 1.61 ; Mmz: 58.62 ± 1.89; lower layers, Bz: 80.47 ± 1.16; Mmz: 70.08 ± 1.83; DE-Stim-7wME, upper layers, Bz: 63.99 ± 1.91 ; Mmz: 34.72 ± 1.90; lower layers, Bz: 58.57 ± 1.61; Mmz: 40.78 ± 1.14). *P ≤ 0.05, **P ≤ 0.001, ***P ≤ 0.0001

We could previously show that the cross-modal recovery becomes apparent from 3 weeks post ME on, following the initial open eye potentiation phase (Van Brussel et al. 2011). We therefore included a condition in which we stimulated SST-interneurons at this later time point (Figs. 1, 8a). Interestingly, the activity pattern appears more similar to sham-7wME, especially in infragranular layers (Fig. 8c, d), and thus revealed higher activity levels than Stim-7wME (Figs. 4b, 8c, d), indicating that late stimulation of SST-interneurons mainly affects cross-modal plasticity limited to supra- and granular layers of the medial monocular cortex (Fig. 8c).

We also combined DE-pretreatment with this late stimulation at 3wME to test the importance of time locking DE and SST-interneuron activation (DE-7wME-Stim at 3w, Figs. 1, 8b). Again, the reactivation appeared stronger compared to the DE-Stim-7wME condition (Fig. 4d). The DE and SST-interneuron activation strategies thus need to coincide in time to accomplish an additive effect in blocking long-term cortical reactivation. In conclusion, SST-interneuron stimulation with or without DE, specifically at the onset of the sensory deprivation, most effectively prevents cross-modal plasticity to reactivate the visual cortex over a period of 7 weeks.

Quantitative comparison of the *zif268*-related OD-values of the early, pre-ME, stimulation-conditions to late stimulation-conditions confirmed a cortical layer-specific effect in reactivation. In the conditions without DE (Fig. 8c, d), late stimulation (7wME-Stim at 3w), resulted in a recovery pattern that is intermediate to sham-7wME and Stim-7wME mice in supra- and granular layers, but similar to sham-7wME mice in infragranular layers. The treatment strategy before or after ME (Sham, SST-interneuron stimulation before ME or at 3wME) has a significant effect on *zif268* expression (One-way ANOVA, upper layers, Bz: *P* = 0.008; Mmz: *P* = 0.005; lower layers, Bz: *P* = 0.016; Mmz: *P* = 0.026). Multiple comparisons *post hoc* tests confirmed that in supra- and granular layers of the Bz, 7wME-Stim at 3w was not different from Sham-7wME or Stim-7wME, but in Mmz, *zif268*-levels of 7wME-Stim at 3w were significantly lower than Sham-7wME. In infragranular layers, 7wME-Stim at 3w revealed significantly higher *zif268*-expression levels compared to Stim-7wME in Bz, but not in Mmz (Sham-7wME versus Stim-7wME, upper layers, Bz: *P* = 0.006; Mmz: *P* = 0.020; lower layers, Bz: *P* = 0.011; Mmz: *P* = 0.022; Stim-7wME versus 7wME-Stim at 3w, upper layers, Bz: *P* = 0.345; Mmz: *P* = 0.962; lower layers, Bz: *P* = 0.007; Mmz: *P* = 0.211; Sham-7wME versus 7wME-Stim at 3w, upper layers, Bz: *P* = 0.084; Mmz: *P* = 0.042; lower layers, Bz: *P* = 0.826; Mmz: *P* = 0.426; Fig. 8e, f).

In conditions with DE (Fig. 8g, h), stimulating SST-interneurons at 3wME (DE-7wME-Stim at 3w) resulted in significantly higher OD-values compared to DE-Stim-7wME cases, both in supra- and granular as well as in infragranular layers (Fig. 8i, j). The treatment strategies before and after ME (Sham, SST-interneuron stimulation before ME combined with DE or at 3 weeks post DE-ME) again had a significant effect on *zif268* expression (One-way ANOVA, upper layers, Bz: *P* < 0.001; Mmz: *P* < 0.001; lower layers, Bz: *P* < 0.001; Mmz: *P* < 0.001; multiple comparisons *post hoc* tests: Sham-7wME versus DE-Stim-7wME, upper layers, Bz: *P* < 0.001; Mmz: *P* < 0.001; lower layers, Bz: *P* < 0.001 ; Mmz: *P* < 0.001; DE-Stim-7wME versus DE-7wME-Stim at 3w, upper layers, Bz: *P* = 0.002; Mmz: *P* = 0.001; lower layers, Bz: *P* = 0.001; Mmz: *P* < 0.001; Sham-7wME versus DE-7wME-Stim at 3w, upper layers, Bz: *P* = 0.080; Mmz: *P* = 0.084; lower layers, Bz: *P* = 0.979; Mmz: *P* = 0.992; Fig. 8i, j). When SST-interneuron stimulation did not temporally coincide with DE, the DE-phenotype was less pronounced or even absent. Indeed, a direct comparison of 7wME-Stim at 3w to DE-7wME-Stim at 3w revealed no significant difference in supra- and granular and infragranular layers, in either Bz or Mmz (Independent samples *t* test of 7wME-Stim at 3w versus DE-7wME-Stim at 3w, upper layers, Bz: *P* = 0.956; Mmz: *P* = 0.270; lower layers, Bz: *P* = 0.900; Mmz: *P* = 0.333).

In conclusion, late stimulation of SST-interneurons results in a milder phenotype compared to optogenetic priming of SST-interneurons prior to ME. Specifically in the supra- and granular layers of the medial monocular cortex, where cross-modal plasticity is maximally active, late stimulation also resulted in decreased activity levels compared to sham-7wME mice, in a way that is similar to early SST-interneuron stimulation. Late SST-interneuron stimulation can thus also affect the potential for cross-modal plasticity, but in a more restricted way compared to early SST-interneuron activation, possibly because of a decoupling from an optogenetically hampered open eye potentiation.

## Discussion

### SST-interneuron stimulation prevents cross-modal take-over of visually deprived cortex

Modulating adult brain plasticity is a topic of great interest in relation to the development of effective strategies to compensate for sensory loss. Whereas enhancement of plasticity could maximize the impact of the reduced sense, suppression of plasticity may be necessary to treat maladaptive outcomes of plasticity, as with phantom pain (Yanagisawa et al. 2016), or to allow the effective integration of a neuro-electronic device that replaces the lost sense after cross-modal plasticity has recruited the deprived cortex for another sense (Lee et al. 2001; Merabet 2011; Sandmann et al. 2012). Strategies that alter inhibitory levels in the visual cortex have been known to affect cortical plasticity (Sale et al. 2010; Nys et al. 2015), but how specific inhibitory components contribute to these mechanisms remains poorly understood. A focused investigation of SST-interneurons now confirmed their ideal position in the cortical circuit to modulate multisensory and cross-modal interactions, as predicted by their location in infragranular layers and their dendritic-targeting properties (Goldberg et al. 2004; Silberberg and Markram 2007; Murayama et al. 2009; Adesnik et al. 2012). A local, optogenetic increase of SST-interneuron activity during a short period immediately prior to partial vision loss by ME resulted in a visual cortex that was unable to reactivate even many weeks after ME. Especially the medial monocular areas failed to become sensitive to whisker inputs (Van Brussel et al. 2011; Nys et al. 2014, 2015). When SST-interneuron stimulation occurred half-way the recovery period, at 3 weeks post-ME, the outcome was similar but more confined to supra- and granular layers of the medial monocular areas. Temporally defined, selective and localized SST-interneuron activity thus determines the level of cortical plasticity reached upon insult. This interneuron population thus seems to regulate the degree to which intact, adjacent sensory areas can colonize nearby deprived cortical territory upon sensory loss.

Recently, Fu et al. (2015) showed in an adult mouse model of ocular dominance plasticity by lid-suture that silencing of SST-interneuron activity in V1b specifically enhanced the open-eye responses, leaving deprived-eye responses unaltered, which are typically only susceptible to changes in young animals during their critical period for ocular dominance formation (Sato and Stryker 2008). SST-interneuron silencing thus specifically enhanced the adult-type of ocular dominance plasticity (Fu et al. 2015). Our finding that optogenetic SST-interneuron stimulation reduces adult plasticity in V1b is in agreement. The inclusion of medial monocular cortex in our analyses now also indicates a role for this inhibitory cell type in regulating adult cross-modal plasticity, suggesting that inhibitory neuronal circuits may regulate cross-modal plasticity in the same way as adult unimodal, ocular dominance plasticity. The most prominent decrease in reactivation in V1b and V1m, as reached upon SST-interneuron stimulation prior to ME, was in register with the site of SSFO-expression pattern (Fig. 6b), indicating that the effect of SST-interneuron activation stays confined to the stimulation site. This suggests that SST-interneurons could serve as a gate, blocking plasticity at those locations where SST-interneurons have been activated specifically at the onset of sensory loss, and even disturb the plasticity process once it is ongoing.

### Combined cortical priming by SST-interneuron prestimulation and DE results in a more severe loss of cortical reactivation

SST-interneuron activation within V1 alone or dark exposure alone (Nys et al. 2015) limit the takeover of deprived cortical areas by intact sensory modalities. Yet a combined approach can result in a much more pronounced suppression of visual cortex reactivation (Figs. 4, 5, 6). Coincident DE and SST-interneuron stimulation pretreatment results in a strongly reduced reactivation profile beyond V1, throughout the entire deprived visual cortex, including the medial monocular regions such as V2M. Moreover, DE also suppresses the cross-modal recruitment of the nearby intact non-visual cortical areas such as the somatosensory barrel field cortex, leading to a lower level of cross-modal invasion and thus lower overall levels of reactivation in the deprived visual cortex (Goel et al. 2006; Van Brussel et al. 2011; Nys et al. 2015). The reported DE-induced re-expression of endocannabinoid receptor-dependent inhibitory long-term depression in adult ocular dominance plasticity (Huang et al. 2010), is likely not linked to SST-interneurons because these receptors are so far not found on this interneuron type (Zeisel et al. 2015). SST-interneurons within the visual cortex are generally silent when the animal is exposed to darkness (Pakan et al. 2016). Other studies looking into experience-dependent cortical plasticity following visual deprivation strategies like DE have pointed towards a mechanism involving PV-interneurons, such as a breakdown of perineuronal nets, importantly situated around PV-interneurons, following a period of DE and subsequent light re-exposure (Murase et al. 2017). In the context of cross-modal plasticity in adult mice, molecular studies previously revealed that increased levels of GABA_A_α1 receptor subunits during DE are correlated with less cross-modal recruitment (He 2006; Nys et al. 2015). This α1 receptor subunit is mainly opposed to fast-spiking PV-positive basket cells (Klausberger et al. 2002), suggesting that PV-interneurons take an important part in regulating cross-modal plasticity processes. The results discussed in this study now add SST-interneurons as important factors controlling the extent of cross-modal plasticity in the visual cortex. Current knowledge about DE and SST-interneurons thus suggests that these two interventions are more likely to employ independent and additive mechanisms to affect cortical plasticity. SST-interneurons, acting as local hubs within the deprived cortex, combined with the decreased recruitment of the intact senses by DE, can in this way maximally shut down the remaining cross-modal input along the full anteroposterior and mediolateral extent of the visual cortex. More precise, with the dendritic-targeting strategy of SST-interneurons (Silberberg and Markram 2007; Kapfer et al. 2007), dendritic inhibition on its own is not powerful enough to block all excitatory signals (Yang et al. 2016), but in combination with a lowered recruitment of intact sensory inputs through DE and the absence of the dominant modality, dendritic inhibition may suffice to gate-off any remaining multisensory input, adding to the notion that SST-interneurons could be highly important modulators of cross-modal input at the level of single excitatory dendrites, which can serve as powerful and specific units in regulating multisensory processing (Chiu et al. 2013; Yang et al. 2016). The DE-induced effect is no longer additive when SST-interneuron stimulation occurs unsynchronized in the middle of the ME recovery period. The reactivation then reaches levels comparable to sham-7wME mice, indicating that the time window opportune to jointly and maximally block cross-modal plasticity lies in earlier stages of the ME-period. Future work is necessary to further explore the mechanism behind the link between DE- and SST-interneuron-related mechanisms. In all, our results indicate that for now the most effective strategies for cross-modal cortical reprogramming are to be enrolled early on, at the onset of sensory loss.

### Possible mechanism of SST-interneuron mediated inhibitory regulation of plasticity

A global increase in inhibition driven by SST-interneurons could also serve as a homogeneous “blanket of inhibition” (Karnani et al. 2014; Jiang et al. 2015), considering their dense connectivity with nearby pyramidal neurons (Fino and Yuste 2011). This could lower overall cortical activity as measured with *zif268*-mRNA, yet is a less likely explanation for the observed effects because SST-interneurons were not optogenetically activated throughout the entire 7wME period, or during the entire late cross-modal phase (week 4–7). It remains to be studied if, and which, chronic effects and which plasticity mechanisms are elicited by the short-term SST-interneuron stimulation resulting in the reduced long-term cortical reactivation profile. It could be that a short-term increase in SST-interneuron-specific inhibition is sufficient to drive the cortical network into a new network state determined by an altered excitation/inhibition balance, which could be related to inducing homeostatic plasticity mechanisms. It remains unclear however, how SST-interneuron stimulation contributes to these processes. Previously, PV-interneurons but not SST-interneurons were found to be involved in activity-dependent matching of inhibition on excitatory neurons in supragranular layers of the mouse visual cortex (Xue et al. 2014). A homeostatic decrease in excitatory transmission is thus unlikely the sole explanation for the lowered reactivation profile after 7wME.

### Circuit level of SST-interneuron modulation of plasticity

**Fig. 9:**
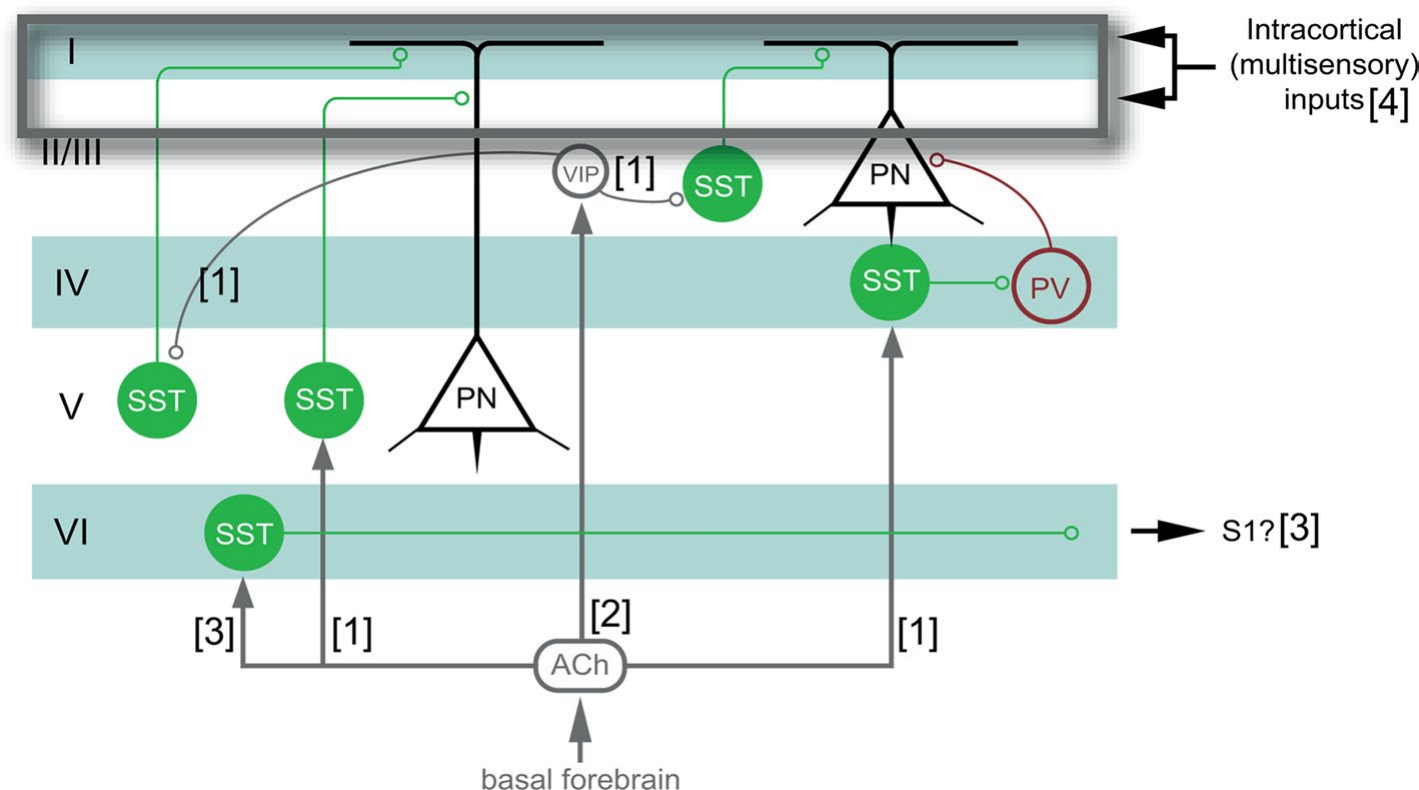
Schematic representation of the possible network circuit by which the VIP-SST-PV-pyramidal neuron circuit could modulate cross-modal inputs into a deprived cortical area. By activating SST-interneurons (green), previously subthreshold multisensory feedback arriving at distal dendrites of pyramidal neurons, could be blocked, preventing a switch from subthreshold to suprathreshold, and impeding cross-modal reactivation of the ME-deprived visual cortex. Grey components indicate modulatory elements (VIP-interneurons and cholinergic signaling through ACh), in a natural condition, which are now circumvented by optogenetic stimulation of SST-interneurons. [1]: Muñoz et al. 2017, [2]: Fu et al. (2015), [3]: Demars and Morishita (2014), [4]: Petrus et al. (2014)

This study directly modulated SST-interneuron activity, leaving the question of how SST-interneurons are integrated within the cortical circuit to gate cross-modal or unimodal plasticity in a natural situation. The disinhibitory VIP-SST-pyramidal neuron circuit motif has been previously implicated in cortical integration processes (Lee et al. 2013), pathway-selective gating (Yang et al. 2016), and adult cortical plasticity (Fu et al. 2014, 2015). Supragranular VIP-interneurons receive long-range corticocortical inputs originating from other cortical areas (Lee et al. 2013), including other primary sensory areas (Stehberg et al. 2014), and cholinergic input related to the behavioral state and the occurrence of salient sensory stimuli (Letzkus et al. 2011). VIP-interneurons may thus be ideally situated to pass on cross-modal input into the cortical (VIP-SST-pyramidal neuron) circuit (Prönneke et al. 2015). Activating SST-interneurons directly, and surpassing the VIP-relay station, could thus inhibit downstream pyramidal neurons at their distal dendrites, as such lowering cortical activity and blocking cross-modal input (Fig. 9). In a natural situation SST-interneurons are also innervated locally by pyramidal neurons (Silberberg and Markram 2007), SST-interneurons could thus act as central hubs between suprathreshold input from the dominant visual modality, and long-range, corticocortical cross-modal input modulated via the VIP-circuit. SST-interneurons also broadly innervate several classes of inhibitory interneurons (Jiang et al. 2015), in particular fast-spiking PV-interneurons (Pfeffer et al. 2013) that somatically inhibit pyramidal neurons (Markram et al. 2004) (Fig. 9). This additional level of disinhibitory control over excitatory drive has been suggested to improve mechanisms of gating selectivity (Yang et al. 2016). The VIP-SST-PV inhibitory circuit may therefore be well organized to control the extent of cross-modal reorganization.

In addition to VIP-interneurons, cholinergic input also directly modulates SST-interneurons through basal forebrain released acetylcholine (ACh) binding to their muscarinic receptors. In S1, VIP-interneurons and ACh differentially regulate the activity of distinct subsets of SST-interneurons during periods of sensory input. VIP-interneurons inhibit specific SST-interneurons, whereas ACh activates other SST-interneurons, imposing an extra level of cortical circuit regulation during sensory processing (Muñoz et al. 2017). Deep SST-interneurons could also impinge on a different circuit mechanism, such as long-range corticocortical connections bridging distinct primary sensory areas (Demars and Morishita 2014) (Fig. 9), and small numbers of pyramidal neurons that respond to sensory inputs other than the one they are dominantly ascribed to in the cortical area in which they reside (Iurilli et al. 2012). In our study, we modulated the activity of all SST-interneurons in the same direction, which potentially biased our interpretation of specific interneuron function in regulating cortical plasticity. Indeed, late stimulation of SST-interneurons resulted in a differential reactivation response in supra- and granular versus infragranular layers. Future work should thus zoom in on the distinct input and output sources of SST-interneurons and how layer-specific SST-interneuron subpopulations receive and respond to direct cross-modal inputs.

## Conclusion

The results from this study bring SST-interneurons forward as promising targets within the cortical circuit to locally modulate adult cross-modal plasticity. These findings offer a deeper understanding of how a specific cell type is engaged in specific aspects of the plastic recovery of sensory deprived cortex. As such, more detailed knowledge about the contribution of other cortical cell types will become useful in the context of brain-computer interfacing as a treatment of sensory loss (van Dokkum et al. 2015) and of maladaptive plasticity where cross-modal takeover is not desirable, by contributing to a detailed understanding on how cortical plasticity can be guided into a pre-defined and desired functional outcome.
